# Functional analysis of the protocatechuate branch of the β-ketoadipate pathway in *Aspergillus niger*

**DOI:** 10.1016/j.jbc.2023.105003

**Published:** 2023-07-01

**Authors:** Michael Sgro, Nicholas Chow, Farnaz Olyaei, Mark Arentshorst, Nicholas Geoffrion, Arthur F.J. Ram, Justin Powlowski, Adrian Tsang

**Affiliations:** 1 Department of Biology, Concordia University, Montreal, Quebec, Canada; 2 Centre for Structural and Functional Genomics, Concordia University, Montreal, Quebec, Canada; 3 Department of Chemistry and Biochemistry, Concordia University, Montreal, Quebec, Canada; 4 Institute of Biology Leiden, Microbial Sciences, Leiden University, Leiden, The Netherlands

**Keywords:** *Aspergillus*, fungi, gene knockout, enzyme kinetics, catabolism, transcriptomics, β-ketoadipate, protocatechuic acid, 3,4-dihydroxybenzoic acid

## Abstract

Bacteria and fungi catabolize plant-derived aromatic compounds by funneling into one of seven dihydroxylated aromatic intermediates, which then undergo ring fission and conversion to TCA cycle intermediates. Two of these intermediates, protocatechuic acid and catechol, converge on β-ketoadipate which is further cleaved to succinyl-CoA and acetyl-CoA. These β-ketoadipate pathways have been well characterized in bacteria. The corresponding knowledge of these pathways in fungi is incomplete. Characterization of these pathways in fungi would expand our knowledge and improve the valorization of lignin-derived compounds. Here, we used homology to characterize bacterial or fungal genes to predict the genes involved in the β-ketoadipate pathway for protocatechuate utilization in the filamentous fungus *Aspergillus niger.* We further used the following approaches to refine the assignment of the pathway genes: whole transcriptome sequencing to reveal genes upregulated in the presence of protocatechuic acid; deletion of candidate genes to observe their ability to grow on protocatechuic acid; determination by mass spectrometry of metabolites accumulated by deletion mutants; and enzyme assays of the recombinant proteins encoded by candidate genes. Based on the aggregate experimental evidence, we assigned the genes for the five pathway enzymes as follows: *NRRL3_01405* (*prcA*) encodes protocatechuate 3,4-dioxygenase; *NRRL3_02586* (*cmcA*) encodes 3-carboxy-*cis,cis*-muconate cyclase; *NRRL3_01409* (*chdA*) encodes 3-carboxymuconolactone hydrolase/decarboxylase; *NRRL3_01886* (*kstA*) encodes β-ketoadipate:succinyl-CoA transferase; and *NRRL3_01526* (*kctA*) encodes β-ketoadipyl-CoA thiolase. Strain carrying Δ*NRRL3_00837* could not grow on protocatechuic acid, suggesting that it is essential for protocatechuate catabolism. Its function is unknown as recombinant NRRL3_00837 did not affect the *in vitro* conversion of protocatechuic acid to β-ketoadipate.

Soil fungi and bacteria play an important role in the carbon cycle by recycling aromatic compounds that are produced in large quantities by plants. Lignin is a complex polymer of aromatic compounds and one of the three major components of lignocellulose along with cellulose and hemicellulose. Lignin is the second most abundant polymer on Earth, after cellulose, accounting for approximately 30% of all organic carbon (1). With global demand for alternative energy, fuels, and chemicals increasing, the availability, cheap cost, and carbon neutrality of lignocellulosic biomass make it a very attractive option (2, 3). Currently, most biorefineries focus on the cellulose and hemicellulose fractions, while using lignin for combustion to meet internal energy demands. However, this use of lignin has low value and only requires about 40% of the total lignin produced (3, 4). To make lignocellulosic biorefineries more economically sustainable, it will be essential to find uses for lignin with more added value (5). For this reason, lignin depolymerization and valorization have become increasingly important and common areas of research (6, 7).

In nature, lignin is degraded mainly by white-rot basidiomycete fungi. These fungi use extracellular oxidoreductases, such as lignin peroxidases and manganese peroxidases, to depolymerize lignin into mono-, di-, and oligo aromatic compounds (8). Another group of fungi, the brown-rot basidiomycete fungi are able to partially oxidize lignin, but not fully degrade it, using non-enzymatic Fenton chemistry (9). Although not as well-studied as lignin degradation by fungi, some bacteria are known to degrade lignin using extracellular oxidative enzymes (10).

Many other aromatic compounds are found in plant biomass. Tannins, the second most abundant group of aromatic compounds in plants, are found in most plant species and tissues and can accumulate in large quantities (11). The hydrolyzable tannins can be oxidatively depolymerized by both bacteria and fungi using tannases (12). The plant metabolites shikimate and quinate are abundant in many plants, comprising around 10% of the dry weight of leaf litter (13) and are used as precursors of aromatic amino acids and their derivatives. Thousands of aromatic compounds are also found linked to polysaccharides or free in the cell, including phenylpropanoids, flavonoids, and coumarins (11, 14).

In aerobic bacteria and fungi, nearly all plant-derived aromatic compounds that can be utilized as a carbon source are funneled into one of seven dihydroxylated aromatic intermediates, which then undergo ring fission and conversion to TCA cycle intermediates (15, 16). These seven intermediates are protocatechuic acid, catechol, hydroxyquinol, gentisic acid, gallic acid, hydroquinone, and pyrogallol (16). Depolymerization of lignin produces compounds derived from the monolignols: sinapic acid, ferulic acid, *p*-coumaric acid, and related compounds (17). Catabolism of these compounds occurs mainly through the intermediates protocatechuic acid (also known as 3,4-dihydroxybenzoic acid, or 3,4-DHB) and catechol (11, 17).

Following conversion to 3,4-DHB, catechol, or other common intermediates, specific metal ion-dependent ring cleavage dioxygenases activate and insert both atoms of O_2_ into the ring fission substrate, all of which are hydroxylated at two positions either *ortho* or *para* to each other (18). The 3,4-DHB and catechol catabolic pathways use separate but analogous sets of enzymes and intermediates to converge on β-ketoadipate, which is further cleaved to succinyl-CoA and acetyl-CoA (19).

Biochemical studies in *Pseudomonas* spp. determined the compounds and enzymes involved in the β-ketoadipate pathway (20, 21) (Fig. 1). Four enzymes were found to convert 3,4-DHB to β-ketoadipate: protocatechuate 3,4-dioxygenase, which cleaves 3,4-DHB to form 3-carboxy-*cis,cis*-muconic acid; 3-carboxy-*cis,cis*-muconate cycloisomerase, which converts 3-carboxy-*cis,cis*-muconic acid into 4-carboxymuconolactone; 4-carboxymuconolactone decarboxylase, which converts 4-carboxymuconolactone to β-ketoadipate enol-lactone; and β-ketoadipate enol-lactonase, which converts β-ketoadipate enol-lactone to β-ketoadipate. Two further reactions convert β-ketoadipate into β-ketoadipyl-CoA then succinyl-CoA and acetyl-CoA, catalyzed by β-ketoadipate-CoA transferase and β-ketoadipyl-CoA thiolase, respectively (20, 22). The genes encoding these enzymes (*pcaA, pcaB, pcaC, pcaD, pcaE,* and *pcaF*) were shown to be part of one or more gene clusters that encode all six enzymes and a transcriptional activator. Fragments of DNA containing these clusters, when expressed, have the ability to fully degrade 3,4-DHB into TCA cycle intermediates (23, 24). In *Acinetobacter baylyii* (formerly identified as *Acinetobacter calcoaceticus*), mutations in the pathway genes resulted in the loss of corresponding enzyme activity (25). Later studies identified two separate genes encode subunits for the bacterial protocatechuate 3,4-dioxygenase (renamed *pcaHG*) and for β-ketoadipate-CoA transferase (renamed *pcaIJ*) (26, 27).

**Figure 1:**
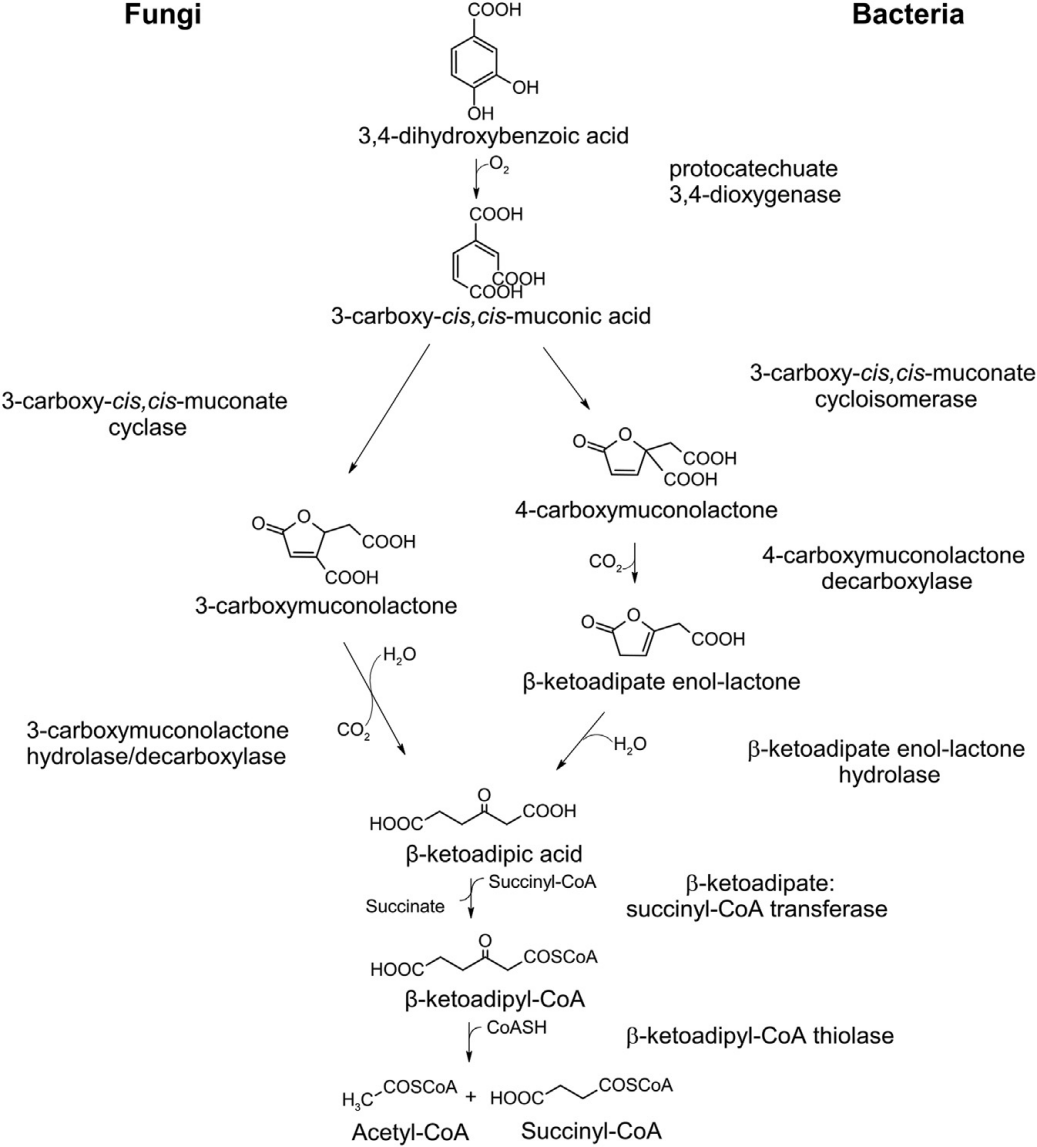
**Summary of the 3,4-dihydroxybenzoic acid catabolic pathway in fungi and bacteria**.

The β-ketoadipate pathway has been studied biochemically in the filamentous fungi *Aspergillus niger* and *Neurospora crassa* (28, 29). The fungal pathway was found to differ from the bacterial pathway in two steps. Following conversion to 3-carboxy-*cis,cis*-muconic acid, fungi convert this compound to 3-carboxymuconolactone instead of 4-carboxymuconolactone, then use a single enzyme with both decarboxylase and hydrolase activity to convert 3-carboxymuconolactone into β-ketoadipate (28, 29) (Fig. 1). Few pathway enzyme activities had been linked to genes in fungi. In a study using two-dimensional gel electrophoresis to determine the differential accumulation of proteins when grown on benzoate (which is catabolized through 3,4-DHB) and sequence homology of those proteins to bacteria, Martins *et al*. (30) predicted four of the five genes involved in the β-ketoadipate pathway in *Aspergillus nidulans*: *AN8566* for protocatechuate-3,4-dioxygenase, *AN1151* for 3-carboxy-cis,cis-muconate cyclase, *AN5232* for 3-carboxymuconolactone hydrolase/decarboxylase, and *AN10495* for β-ketoadipate:succinyl-CoA transferase. Mutants lacking *AN1151* and *AN5232* genes grew poorly on benzoate and accumulated compounds that appeared to be 3-carboxy-*cis,*cis-muconic acid and 3-carboxymuconolactone based on absorption spectra (30), confirming their involvement in benzoate utilization. The assignment of AN8566 as protocatechuate-3,4-dioxygenase is supported by mutational and biochemical analysis of its orthologue, NRRL3_01405, in *A. niger* (31, 32). However, the assignment of AN5232 as the 3-carboxymuconolactone hydrolase/decarboxylase is inconsistent with the earlier results of Thatcher and Cain (33) who determined the mass of this purified enzyme in *A. niger* to be 54 kDa while AN5232 has a mass of 26 kDa. Hence to date, our knowledge of the genes involved in the β-ketoadipate pathway in fungi remains incomplete.

The filamentous fungus *A. niger* is an important cell factory for the production of enzymes and organic acids (34). The genome of *A. niger* NRRL3 is the only publicly available fungal genome that has been fully curated by biocurators, an important reference for genome-wide studies (35). In this study, we examined the catabolic pathway of protocatechuate of *A. niger*. We have used data from sequence homology, transcriptomes, enzyme activity, and the characterization of growth phenotype of knockout mutants in combination with metabolite analysis to unambiguously determine the enzymes involved in each step in the catabolism of protocatechuate in *A. niger*. The knowledge gained by the characterization of these enzymes may lead to the valorization of lignin-derived bioactive compounds in *A. niger* and other fungi (36, 37). As the compounds produced by lignin degradation in fungi are different from those produced by bacteria, filamentous fungi like *A. niger* present different potential options for lignin valorization (36).

## Results

### Prediction of genes involved in 3,4-dihydroxybenzoic acid (3,4-DHB) utilization in *A. niger* based on orthology and transcriptomics

To identify the genes involved in the catabolism of 3,4-DHB in *A. niger*, we first made predictions based on genes previously implicated in *A. nidulans* and *A. niger* (30, 31) and homology to genes in other fungi and bacteria. The sequence comparisons resulted in multiple gene candidates being considered for four of the five enzymes in the pathway. We then used comparative transcriptomics to provide experimental evidence in support of the candidate genes (Table 1). We performed RNA sequencing using *A. niger* grown by batch fermentation in a bioreactor with media containing either fructose or 3,4-DHB as the sole carbon source. Growth of *A. niger* on 3,4-DHB was relatively poor with a maximum growth rate during exponential growth of 0.0844 ± 0.0041 (g dry weight/kg broth/h) and maximum biomass accumulation (3.3015 ± 0.007 dry weight/kg broth compared to growth rate and biomass accumulation on fructose (0.217 ± 0.002 g dry weight/kg broth/h) and maximal biomass accumulation (4.289 ± 0.194 g dry weight/kg broth). To minimize the effects on global gene expression caused by differential growth rates, we performed a separate RNA sequencing experiment using *A. niger* grown in shake flasks containing complete media followed by a transfer of mycelia into media containing either fructose or 3,4-DHB as the sole carbon source. Transcriptome results of the candidate pathway genes from the transfer cultures are shown in Table 1, those from the bioreactors are shown in Table S2, and the complete transcriptome datasets from both fermentations and their analysis are presented in Tables S3 and S4. In both the bioreactor and transfer culture samples, clear primary candidates were observed for four of the five enzymes based on the level of expression in the 3,4-DHB media and upregulation in that media compared to the fructose media.

**Table 1:** RNA sequencing data from transfer culture for genes with homology to 3,4-DHB pathway genes

Enzyme	Predicted gene	Mean fructose TPM	Mean 3,4-DHB TPM	Fold change (3,4-DHB vs. Fructose)
Protocatechuate 3,4-dioxygenease	***NRRL3_01405***	**8.24**	**18,330.48**	**2223.40**
	*NRRL3_02644*	16.77	464.59	27.70
	*NRRL3_04277*	1.62	0.72	0.45
	*NRRL3_04787*	11.46	100.19	8.74
	*NRRL3_05330*	2.64	9.31	3.53
Carboxy-cis,cis-muconate cyclase	***NRRL3_02586***	**16.88**	**1016.83**	**60.23**
3-carboxymuconolactone hydrolase/decarboxylase	*NRRL3_00837*	21.48	1038.39	48.34
	***NRRL3_01409***	**10.51**	**727.89**	**69.23**
	*NRRL3_03759*	1.51	6.76	4.49
	*NRRL3_08340*	15.20	3661.57	240.94
β-ketoadipate:succinyl-CoA transferase	*NRRL3_01593*	32.52	55.19	1.70
	***NRRL3_01886***	**20.66**	**1499.22**	**72.58**
	*NRRL3_11640*	32.14	360.56	11.22
β-ketoadipyl-CoA thiolase	***NRRL3_01526***	**19.21**	**1410.97**	**73.47**
	*NRRL3_07786*	274.13	254.02	0.93
	*NRRL3_11162*	37.38	143.40	3.84

Mean values are the average of duplicates. Complete data and DESeq2 (58) analysis shown in Table S3. Predicted genes in bold. TPM, transcripts per million.

Catabolism of these compounds begins with a ring-opening step catalyzed by an intradiol ring cleavage dioxygenase. In the *A. niger* NRRL3 genome, five gene models have been annotated as non-secreted intradiol ring cleavage dioxygenases, based on homology to other fungi and bacteria (32). Among the five genes predicted to encode non-secreted intradiol ring cleavage dioxygenases, *NRRL3_01405* was the most highly upregulated, >2000-fold (Table 1), and was the third most highly expressed gene in the transfer culture transcriptome in the 3,4-DHB media (Table S3).

Previous studies have identified and purified an enzyme with 3-carboxy-*cis,cis*-muconate cyclase activity from *p*-hydroxybenzoate-grown *A. niger.* Although this enzyme was characterized quite extensively, no corresponding gene has been unambiguously identified (28, 38, 39). Only one gene in the NRRL3 genome, *NRRL3_02586,* is homologous with the characterized *N. crassa* carboxy-cis,cis-muconate cyclase CMLE_NEUCR (40), which exhibits 67% sequence identity with NRRL3_02586. This gene was highly expressed and upregulated ∼60-fold in 3,4-DHB media (Table 1).

Cain and co-workers (28) established that 3-carboxymuconolactone is converted in a single step to β-ketoadipate by a 54 (±5) kDa *A niger* enzyme with both hydrolase and decarboxylase activities, but no gene has been identified subsequently. This is in contrast to bacteria, where 3-carboxymuconolactone is instead lactonized to 4-carboxymuconolactone and converted first by a decarboxylase, then a hydrolase, to β-ketoadipate (19) (Fig. 1). In *A. nidulans*, Martins *et al*. (30) identified a gene encoding a 26 kDa protein, AN5232, weakly induced in the proteome by growth on benzoate, and with weak similarity to 4-carboxymuconolactone decarboxylase, that when knocked out abolished growth on benzoate and resulted in accumulation of all intermediates up to 3-carboxymuconolactone. On this basis, AN5232 was assigned the function of 3-carboxymuconolactone hydrolase (decarboxylating) (30). The *A. niger* orthologue of AN5232 is NRRL3_00837 (72% identity), which was found to be upregulated ∼48-fold in the transcriptome in response to 3,4-DHB (Table 1). However, sequence comparisons and protein domain analysis provide no clue that *NRRL3_00837* encodes hydrolase activity, and its predicted molecular weight (25.48 kDa) is inconsistent with the 54-kDA protein that has been reported for 3-carboxymuconolactone decarboxylase/hydrolase in *A. niger* (33).

In an effort to identify more potential candidates for 3-carboxymuconolactone hydrolase (decarboxylating), the sequences of various characterized bacterial lactone hydrolases and decarboxylases from aromatic pathways were used in BLASTP searches of the NRRL3 genome. Using the sequence of ELH2_ACIAD, a 3-oxoadipate enol-lactonase from *A. baylyii* (41), NRRL3_01409, with 28% identity and containing an alpha/beta hydrolase fold-1 domain with a predicted molecular weight of 60.95 kDa, appeared to be a good candidate. *NRRL3_01409* is upregulated ∼70-fold on 3,4-DHB (Table 1). Two other genes, *NRRL3_08340* and *NRRL3_03759*, were homologous to carboxymuconolactone decarboxylase-like proteins. *NRRL3_08340* was upregulated ∼240-fold on 3,4-DHB, while *NRRL3_03759* was only minimally upregulated (∼4-fold) in response to 3,4-DHB (Table 1).

For the final two enzymes of the pathway, β-ketoadipate CoA transferase and β-ketoadipyl-CoA thiolase, three potential candidates for each are present in the genome. The β-ketoadipate CoA transferase had one clear candidate, *NRRL3_01886*, which is the most highly upregulated (∼73-fold) on 3,4-DHB (Table 1). Although two other genes, *NRRL3_11640* and *NRRL3_01593*, are also homologous to succinyl-CoA:3-ketoacid coenzyme A transferases (the systematic name of this enzyme), and showed some upregulation, *NRRL3_01886* had a much higher expression level and 3,4-DHB/fructose expression ratio. Similarly, of the three genes that were homologous to 3-ketoacyl-CoA thiolase, *NRRL3_01526* was highly expressed and differentially upregulated on 3,4-DHB (∼73-fold), while *NRRL3_07786* and *NRRL3_11162* were not (Table 1).

### Identification of genes required for 3,4-DHB utilization by gene deletion and metabolomics

**Figure 2:**
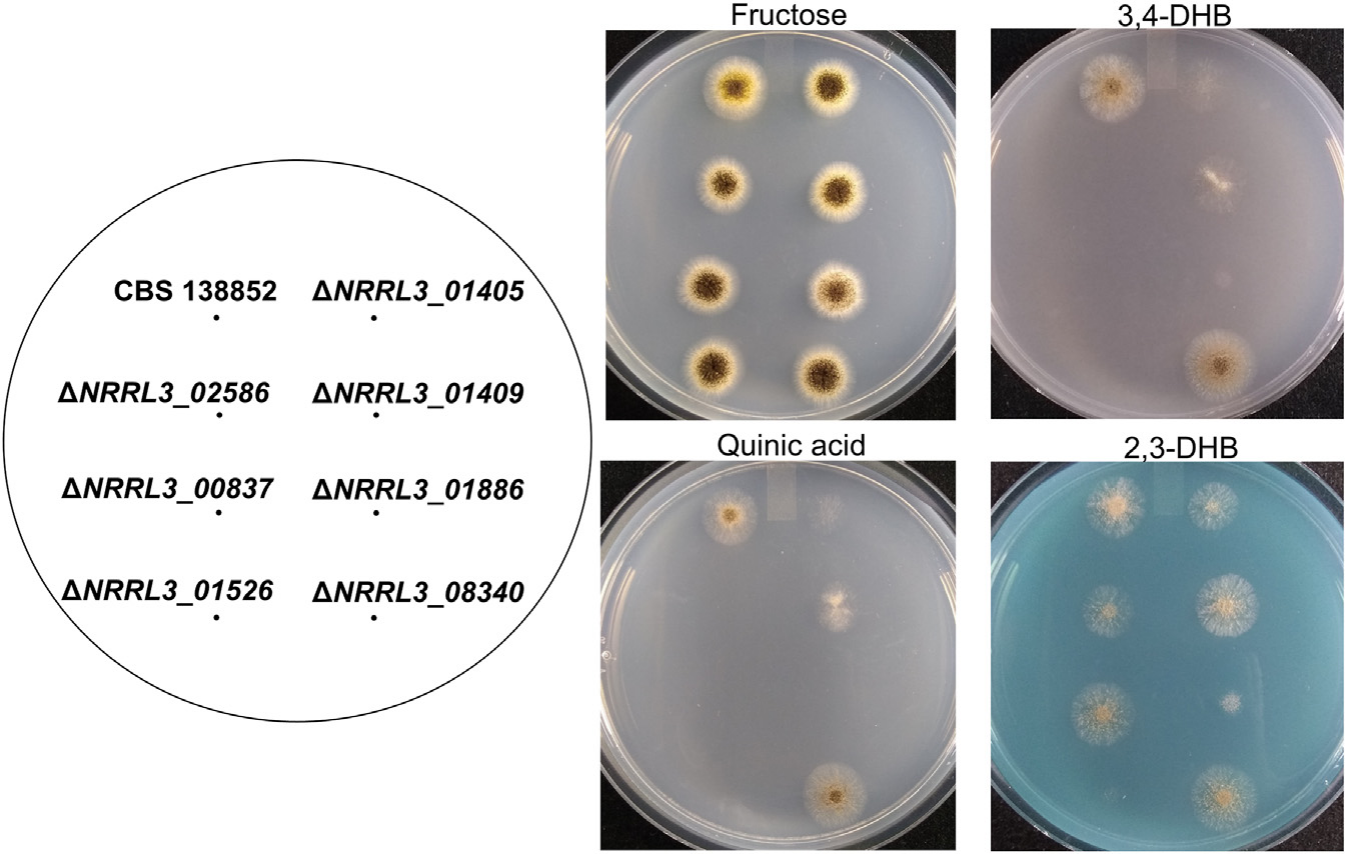
**Growth phenotype of *A. niger* CBS 138852 and predicted 3,4-DHB pathway mutants on various carbon sources (0.5% w/v): fructose, 3,4-DHB, quinic acid and 2,3-DHB.** Plates were incubated at 30 °C for 72 h.

To confirm the involvement of the predicted genes in the 3,4-DHB pathway we generated knock-out mutants in *A. niger* CBS 138852. We grew these mutants on solid media containing fructose, 3,4-DHB, quinic acid (degraded through the 3,4-DHB pathway (13)), and 2,3-DHB (degraded through the catechol branch of the pathway (42)), as sole carbon sources (Fig. 2). Growth experiments were performed three or more times using two or more independently isolated mutant transformants for each gene, with similar results observed in all experiments. We also grew all mutants on fructose media as a control in case of general effects on growth, as these genes have no expected effect on fructose metabolism. All mutants showed an identical growth phenotype on fructose compared to the parental strain, as expected.

The Δ*NRRL3_01405* mutant showed very weak growth on 3,4-DHB and failed to sporulate, further supporting the assignment of this gene as the protocatechuate-3,4-dioxygenase (Fig. 2). As expected, Δ*NRRL3_01405* displayed defective growth on quinic acid, while it had minimal effects on growth on 2,3-DHB.

The predicted carboxy-*cis,cis*-muconate cyclase mutant, Δ*NRRL3_02586*, entirely failed to grow on 3,4-DHB or quinic acid media, while growth on 2,3-DHB media appeared to be slightly impaired (Fig. 2). The growth of this mutant on fructose remained unaffected.

The gene(s) involved in the next step of the pathway, conversion of 3-carboxymuconolactone to β-ketoadipic acid, is unclear. Three of the four candidates predicted for this step displayed differential upregulation when cultured on 3,4-DHB (Table 1). We therefore deleted individually the three upregulated genes: *NRRL3_00837*, *NRRL3_01409*, and *NRRL3_08340*. *NRRL3_08340* is up-regulated ∼240-fold when grown on 3,4-DHB and is an orthologue of the bacterial 4-carboxymuconolactone decarboxylase. However, *ΔNRRL3_08340* did not impair growth on any of the compounds tested (Fig. 2). Growth on 3,4-DHB and quinic acid was abolished for Δ*NRRL3_00837* and severely impaired for Δ*NRRL3_01409* (Fig. 2).

Mutants generated for *NRRL3_01886* and *NRRL3_01526* grew very poorly or entirely failed to grow on all of the compounds tested except fructose (Fig. 2). Based on literature (19), the enzymes encoded by these genes are shared by the multiple branches of the β-ketoadipate pathway.

**Figure 3:**
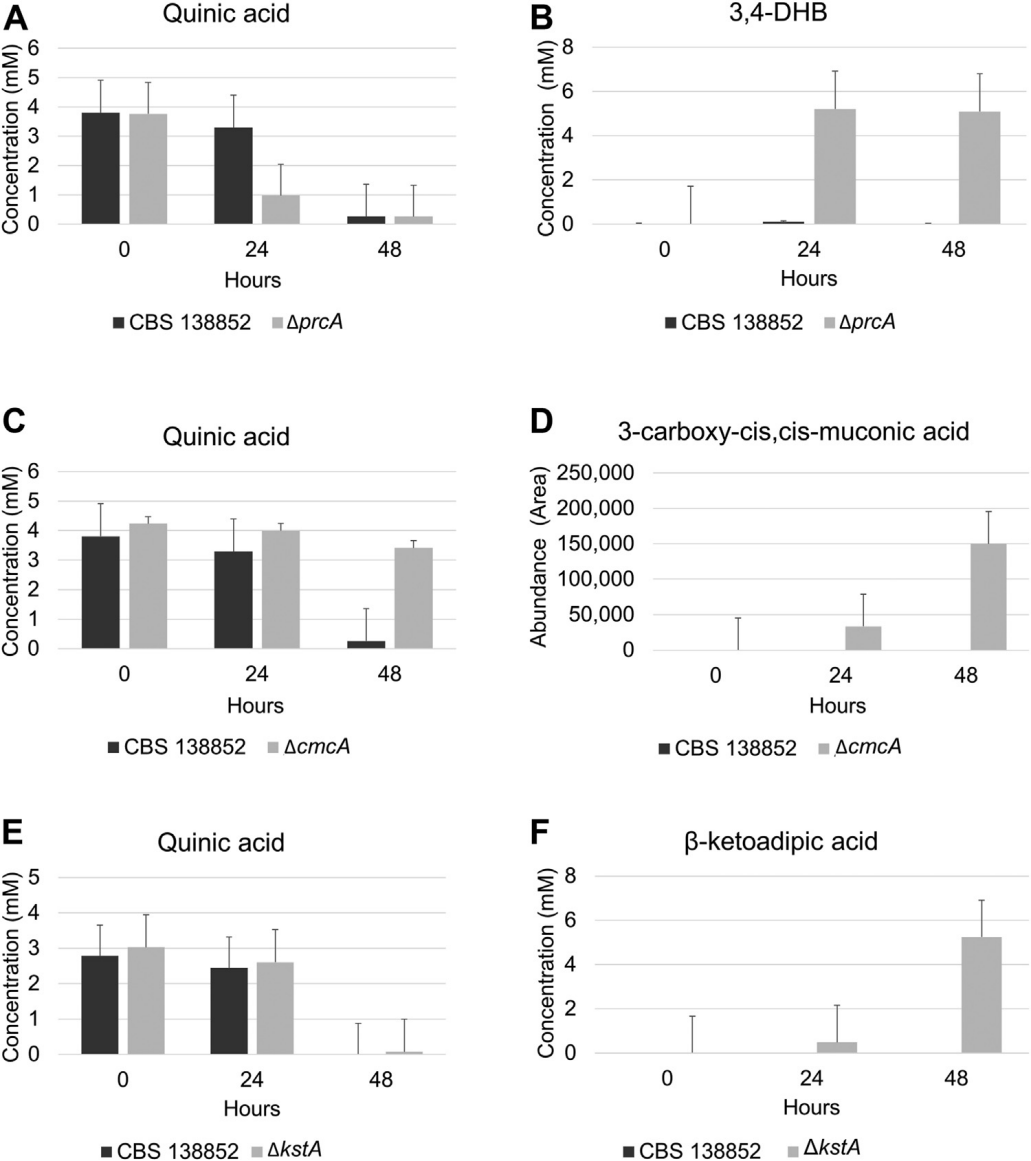
**Accumulation of pathway intermediates in 3,4-DHB pathway mutants in minimal media with quinic acid as sole carbon source using LC-MS.***A*, consumption of quinic acid and (*B*) accumulation of 3,4-DHB in parent and Δ*prcA* strains; (*C*) consumption of quinic acid and (*D*) accumulation of 3-carboxy-*cis,cis-*muconic acid in parent and Δ*cmcA* strains; (*E*) consumption of quinic acid; and (*F*) accumulation of β-ketoadipic acid in parent and *ΔNRRL3_01886* strains. Data from LC-MS in negative mode (*A*–*E*) and positive mode (*F*). Results are the average of triplicate samples for each strain.

Pathway mutants can be expected to accumulate metabolites that correspond to the products of the preceding steps of the pathway (43). We used liquid chromatography-mass spectrometry to examine metabolites accumulated in the extracellular media following the cultivation of pathway mutants on quinic acid. Mutants were grown on complete media and mycelia were transferred to media with quinic acid as the sole carbon source. The degradation of quinic acid in the Δ*NRRL3_01405* mutant (Fig. 3*A*) resulted in extracellular accumulation of 3,4-DHB at much greater levels than were observed in the parent strain (Fig. 3*B*), as expected of a mutant lacking the protocatechuate-3,4-dioxygenase. *NRRL3_01405* has previously been named *prcA* for protocatechuate 3,4-dioxygenase (31). Results from metabolite analysis presented here support the previous conclusion that *NRRL3_01405* encodes protocatechuate 3,4-dioxygenase, we therefore retain the previously assigned gene name and refer to NRRL3_01405 as PrcA.

Although the Δ*cmcA* mutant consumed quinic acid at a much slower rate than the parent strain, accumulation of a compound with mass equal to that of 3-carboxy-*cis,cis*-muconic acid was observed in the mutant using LC-MS (Fig. 3, *C* and *D*). This compound was not detected in the parent strain. No standard of 3-carboxy-*cis,*cis-muconic acid was available; however, MS/MS fragment spectra (Fig. S9) from this compound were similar to those predicted by Agilent MassHunter Molecular Structure Correlator to be 3-carboxy-*cis,cis*-muconic acid. Combined with the observed growth phenotype, these results show that *NRRL3_02586* likely encodes 3-carboxy-*cis,cis*-muconate cyclase. We therefore refer NRRL3_02586 as 3-carboxy-*cis,cis*-muconate cyclase (CmcA) and its encoding gene as *cmcA*.

We were unable to detect 3-carboxymuconolactone accumulation in the Δ*NRRL3_00837* or Δ*NRRL3_01409* mutants using LC-MS (Fig. S10), so we relied on biochemical analysis to determine the activities of the proteins encoded by each of these genes (see below). However, in the Δ*NRRL3_01886* mutant grown on quinic acid, LC-MS detected accumulation of a compound with a mass of 160.01 g/mol, which corresponds to β-ketoadipic acid (monoisotopic mass 160.037173 g/mol), (Fig. 3, *E* and *F*), further confirming that *NRRL3_01886*, which we call *kstA*, encodes the β-ketoadipate:succinyl-CoA transferase. Extracted ion chromatograms of intermediates accumulated in the Δ*prcA*, Δ*cmcA* and Δ*kstA* mutants are compared to the parent strain and to standards of 3,4-DHB (for Δ*prcA*) and β-ketoadipate (for Δ*kstA*) in Fig. S11.

### Biochemical analysis of 3,4-dihydrobenzoic acid (3,4-DHB) pathway enzymes

**Figure 4:**
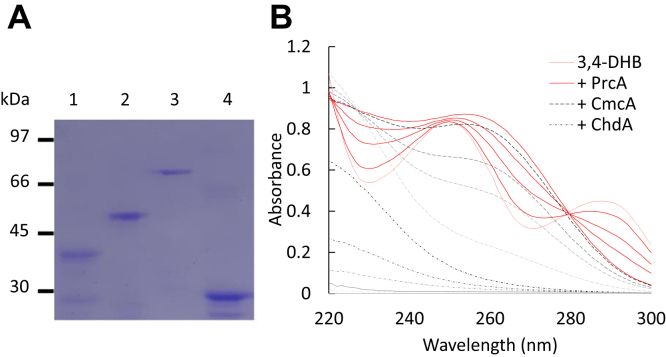
***In vitro* assay of purified pathway enzymes.***A*, SDS-PAGE of purified enzymes used in this assay. Lane 1, NRRL3_01405*;* Lane 2, NRRL3_02568; Lane 3, NRRL3_01409; Lane 4, NRRL3_00837. The main band in each lane was excised and subjected to peptide mass fingerprinting as described in Experimental procedures. *B*, spectral changes accompanying conversion of 3,4-DHB. The initial spectrum is that of 3,4-DHB (100 μM) in buffer, followed by sequential additions of PrcA (NRRL3_01405; 6.35 μg), CmcA (NRRL3_02586; 0.19 μg) and ChdA (NRRL3_01409; 0.38 μg). The time series for PrcA is indicated at the far right, with the initial spectrum obtained after the enzyme indicated by 0, with subsequent scans at 0.15, 1, and 5 min. Time series for +CmcA (*middle*) and +ChdA (*far left*) are similarly indicated. The scan speed was 360 nm/min.

We successfully expressed most of the selected candidate genes described above in *Escherichia coli*, and their recombinant proteins were purified so that they could be used for direct enzymatic assays to confirm molecular function. Figure 4*A* shows the SDS-PAGE of four purified candidate pathway proteins: NRRL3_00837, NRRL3_01405, NRRL3_01409, and NRRL3_02586. The activities of each individual enzyme were assayed by established procedures (28) and as is shown by the spectral changes recorded in Figure 4*B*. The enzymes encoded by NRRL3_01405, NRRL3_02586, and NRRL3_01409 showed the expected activity (28, 39) and were sufficient for the stepwise conversion of protocatechuate to β-ketoadipate.

**Figure 5:**
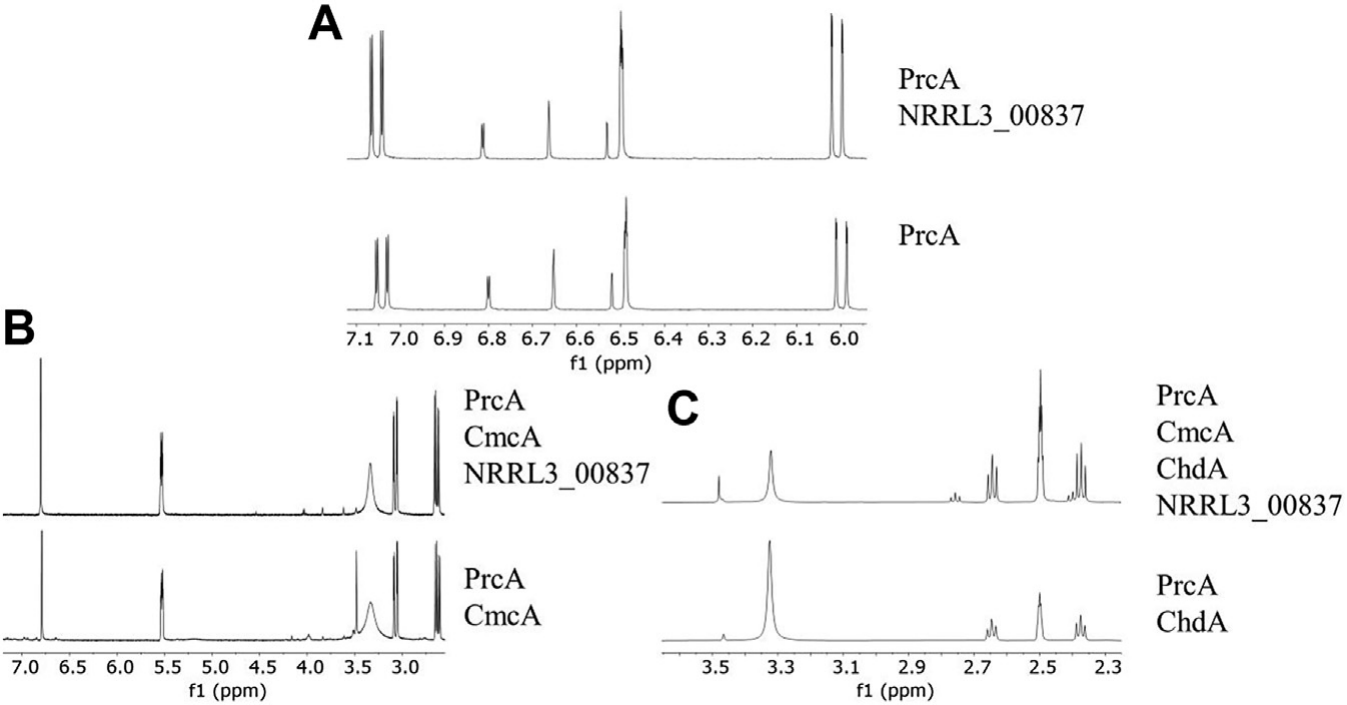
^**1**^**H NMR (500 MHz, DMSO) spectra of the compounds produced from the incubation of protocatechuate withvarious β-ketoadipate pathway enzymes.** (*A*) PrcA (NRRL3_01405) (*lower*), PrcA + NRRL3_00837 (*upper*); (*B*) PrcA + CmcA (NRRL3_02586) (*lower*), PrcA + CmcA + NRRL3_00837 (*upper*); (*C*) PrcA, CmcA + ChdA (NRRL3_01409) (*lower*) PrcA + CmcA + ChdA + NRRL3_00837 (*upper*).

The *NRRL3_01405*-encoded PrcA enzyme was previously shown to be active on 3,4-DHB, but not catechol, converting it to a compound with the spectral properties of 3-carboxy-*cis,cis*-muconate (32). We observed this spectral change again, as expected (Fig. 4*B*). The ^1^H-NMR spectrum of the product (Fig. 5*A*) showed that chemical shifts and coupling constants of 3-carboxy-*cis,cis*-muconate are similar with the exception of multiplicity where Yamanashi *et al*. (44) observed allylic coupling; δ 6.01(d, 1H, J = 11.5 Hz), 6.50 (d,1H, J = 2.0 Hz), 7.05 (dd, 1H, J = 2.0 Hz, 11.5 Hz) *versus* δ 6.01(dd, 1H, J = 0.9, 12.0 Hz), 6.50 (m, 1H), 6.77 (dd, 1H, J = 1.7, 12.0 Hz). Additional peaks at 2.63, 3.06, 5.53 and 6.85 PM are consistent with the presence of some 3-carboxy-*cis*-*cis*-muconolactone (see below). Although no lactonizing enzyme was present, it is likely that some acid-catalyzed lactonization occurred when the sample was prepared for extraction in ethyl acetate. Rapid acid-catalyzed isomerization and lactonization have been reported by others (*e.g.* (45)), which Yamanashi *et al*. (44) avoided by preparing the sample without acidification and extraction. A few minor additional contaminating peaks were not assigned.

The addition of the purified *NRRL3_02586*-encoded CmcA enzyme to a PrcA and 3,4-DHB reaction mixture resulted in further spectral change consistent with the rapid conversion of 3-carboxy-*cis,cis*-muconate to 3-carboxymuconolactone (Fig. 4*B*). ^1^H-NMR analysis of this mixture (Fig. 5*B*, lower panel) showed that the product of PrcA and CmcA was 3-carboxy-*cis*-*cis*-muconolactone by comparing 1H-NMR of muconolactone from Yamanashi *et al*. (44) as the additional peaks and coupling constants are consistent in muconolactone due to the hydrogen at C5: δ 2.63 (dd, 1H, J = 7.9, 16.4 Hz), 3.06 (dd, 1H, J = 3.3, 16.4 Hz,), 5.53 (ddd, 1H, J = 2.1, 3.3, 7.9 Hz), 6.80 (d, 1H, J = 2.1 Hz) *versus* δ 2.71 (dd, 1H, J = 8.2, 16.6 Hz), 2.95 (dd, 1H, J = 4.8, 16.6 Hz), 5.57 (dddd, 1H, J = 1.4, 1.9, 4.8, 8.2 Hz), 6.24 (dd, 1H, J = 1.9, 5.8 Hz), 7.81 (dd, 1H, J = 1.4, 5.8 Hz). The reported chemical shifts are also consistent with 3-carboxymuconolactone values from Kondo *et al*.: δ 2.67, 3.10, 5.55, 6.81 ppm (46).

When we added the protein encoded by *NRRL3_01409* to a reaction mixture of 3,4-DHB with PrcA and CmcA UV spectral features were abolished, as has been reported for the conversion of 3-carboxymuconolactone to β-ketoadipate (28) (Fig. 4*B*). The ^1^H-NMR spectrum of the product (Fig. 5*C*, lower panel) was essentially identical to that of β-ketoadipate as reported by Yamanashi *et al*. (44): δ 2.38 (t, 2H, J = 6.7 Hz), 2.65 (t, 2H, J = 6.7 Hz), 3.48 (s, 2H) *versus* δ 2.55 (t, 2H, J = 6.5 Hz), 2.86 (t, 2H, J = 6.5 Hz), 3.39 (s, 2H). We therefore refer to NRRL3_01409 as 3-carboxymuconolactone hydrolase/decarboxylase and its encoding gene *chdA*.

The observed products were the same when the protein encoded by *NRRL3_00837* was included in the reaction mixtures with PrcA, CmcA, and ChdA shown in Figure 5 (compare upper spectra to lower spectra). In addition, no significantly different spectral changes were detected when the experiment shown in Figure 4 was repeated in the presence of purified NRRL3_00837, nor did it catalyze any one of these steps alone (data not shown). Thus, NRRL3_00837 does not affect the chemistry of the 3,4-DHB to β-ketoadipate conversion. We tested the possibility that the protein encoded by *NRRL3_00837* could enhance the rates of one of the enzymes between PCA and β-ketoadipate by adding this protein at one of two different concentrations to single wavelength assays of each of the three enzymes. As shown in Table 2, none of these rates was significantly affected. Together, these data show that the protein ChdA, encoded by *NRRL3_01409*, is sufficient to catalyze the conversion of 3-carboxymuconolactone to β-ketoadipate, while NRRL3_00837 cannot; furthermore NRRL3_00837 does not affect any of the reaction rates between 3,4-DHB and β-ketoadipate.

**Table 2:** Rates of pathway enzymes in the presence or absence of the protein encoded by NRRL3_00837

Enzyme	Activity (μM/min)
PrcA	78.3 ± 0.75
+ 00,837 (2.45 μg)	82.2 ± 4.14
+ 00,837 (12.25 μg)	83.2 ± 8.20
CmcA	70.2 ± 5.32
+ 00,837 (2.45 μg)	75.2 ± 5.30
+ 00,837 (12.25 μg)	70.1 ± 2.90
ChdA	43.5 ± 3.90
+ 00,837 (2.45 μg)	46.1 ± 1.16
+ 00,837 (12.25 μg)	42.1 ± 5.06

Mean values are the average of triplicates.

Attempts to produce the enzyme encoded by *kstA* (NRRL3_01886) were unsuccessful. Although the protein encoded by *NRRL3_01526* (*kctA*) was produced in recombinant form, without KstA we were unable to generate β-ketoadipyl-CoA to perform the coupled assay to confirm function biochemically.

## Discussion

**Figure 6:**
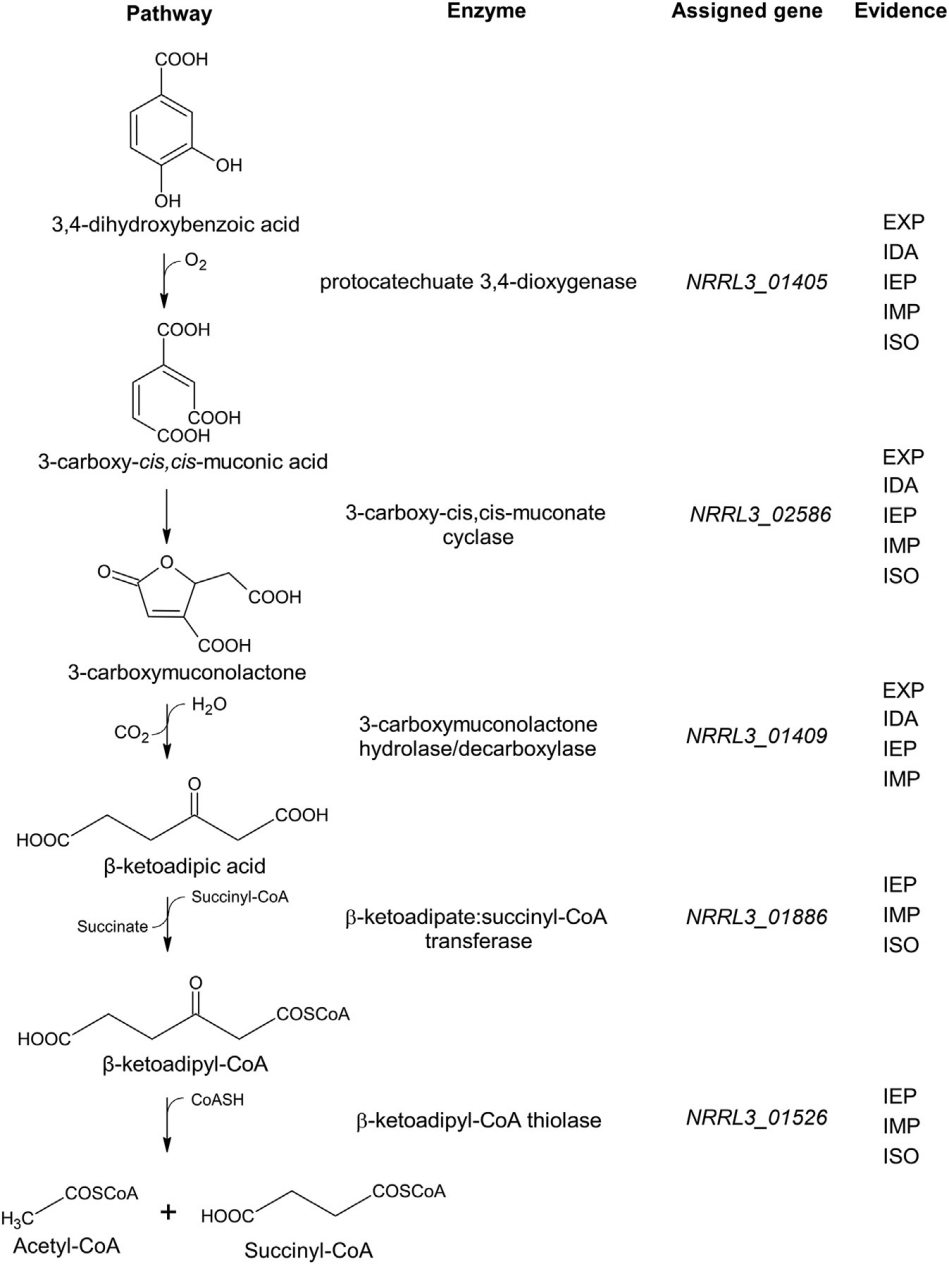
**Summary of the 3,4-dihydroxybenzoic acid catabolic pathway in *A. niger*.** Genes assigned for enzymes in the pathway based on the evidence from various approaches: EXP, inferred from experiment; IDA, inferred from direct assay; IEP, inferred from expression pattern; IMP, inferred from mutant phenotype; ISO, inferred from sequence orthology.

While the biochemistry and molecular biology of the β-ketoadipate pathways are well-characterized in bacteria, in fungi the information we have is partial and often not linked to genes. Conversely, the genomes of fungi are full of sequence annotations that link genes to function in these pathways, with very little supporting evidence. In this study, we provide evidence for the specific gene complement that is involved in the β-ketoadipate pathway for 3,4-dihydroxybenzoate (3,4-DHB) using a combination of homology, transcriptome analysis, mutational analysis, and biochemical characterization. Figure 6 summarizes the genes involved and the available evidence supporting the functional assignments. The supporting evidence is as follows: (1) EXP, inferred from experiment, where mutation in the gene results in the accumulation of metabolite that corresponds to the product of the reaction catalyzed by the enzyme of the preceding step of the pathway; (2) IDA, inferred from direct assay, where the recombinant protein encoded by the gene catalyzes the stated enzyme activity by biochemical assay; (3) IEP, inferred from expression pattern, where the gene is differentially upregulated when cultured on 3,4-DHB; (4) IMP, inferred from mutant phenotype, where strain carrying the mutated gene displays severe growth deficiency on 3,4-DHB; and (5) ISO, inferred from sequence orthology, where orthologues of the gene in related organisms have been characterized to have the stated function.

Sequence similarity to orthologues of characterized enzymes suggested multiple *A. niger* genes are potentially involved in four of the five steps of the 3,4-DHB catabolic pathway (Table 1). Comparison of the transcriptomes of growth on fructose and 3,4-DHB, both those obtained by transfer culture (Table S3) and those obtained by batch fermentation in the bioreactor (Table S4), provided useful clues to determine which of the candidate genes for the other four steps were likely involved in the catabolism of 3,4-DHB. Although both methods of cultivation led to similar results concerning the candidate genes, the transcriptomes had several notable differences. Perhaps due to the reduced exposure time to 3,4-DHB, the transfer culture samples had higher expression levels for each of the genes predicted to be in the pathway. However, compared to the bioreactor samples, many additional genes were also more highly expressed. Filtering the transcriptomes to include only genes with an average TPM on 3,4-DHB of 50 or greater and a fold change of 5 or greater resulted in a list of 430 genes for transfer cultures but only 223 for the bioreactor cultures. Among these genes, 84 appeared on both lists, including one of the candidate genes for each enzyme except for ChdA, 3-carboxymuconolactone hydrolase/decarboxylase, which had three candidates on both lists: *NRRL3_00837*, *NRRL3_01409*, and *NRRL3_08340*. Thus, for each step except that catalyzed by ChdA, the most highly induced candidate was subsequently confirmed by deletion analysis to be required for growth on 3,4-DHB as described below. Many of the remaining genes which appeared on both lists were transporters and transcription factors, though there were no clear candidates for the 3,4-DHB pathway, as well as hypothetical proteins and dehydrogenases. Genes appearing solely in the transfer culture list included several genes annotated as being involved in other aromatic catabolic pathways including homogentisate and 2,3-DHB. The 3,4-DHB transcriptomes in the bioreactor cultures included a higher expression level for biosynthetic gene clusters involved in secondary metabolism; for example, the TAN-1612/BMS-192548 cluster (NRRL3_09545-NRRL3_09550) and the yanuthone D cluster (NRRL3_06287-NRRL3_06296) (47), perhaps a sign of stress as shown by slow growth on 3,4-DHB.

Previously, Martins *et al*. (30) assigned four genes to the 3,4-DHB catabolic pathway in *A. nidulans*. Table 3 lists the *A. niger* proteins involved in 3,4-DHB catabolism, revealed in this study, and their *A. nidulans* orthologues. Our results agreed with three of the gene assignments made by Martins *et al*. (30): AN8566 as protocatechuate 3,4-dioxygenase, AN1151 as 3-carboxy-*cis,cis*-muconate cyclase, and AN10495 as β-ketoadipate:succinyl-CoA transferase. The *A. nidulans* protein AN5232 assigned as 3-carboxymuconolactone hydrolase/decarboxylase is the orthologue of *A. niger* NRRL3_00837. We showed here that NRRL3_00837 is an essential component of the 3,4-DHB catabolic pathway, but its role is currently unresolved. We identified NRRL3_01409 as 3-carboxymuconolactone hydrolase/decarboxylase and NRRL3_01526 as β-ketoadipyl-CoA thiolase, their *A. nidulans* orthologues AN10520 and AN5698 had not been characterized previously.

**Table 3:** *Aspergillus niger* proteins implicated in the 3,4-DHB catabolic pathway by this study compared to their orthologues in *A. nidulans*

3,4-DHB catabolic pathway proteins	*A. niger*	*A. nidulans* orthologue	Sequence identity
Protocatechuate 3,4-dioxygenase	NRRL3_01405	AN8566^a^	94%
3-carboxy-*cis,cis*-muconate cyclase	NRRL3_02586	AN1151^a^	91%
3-carboxymuconolactone hydrolase/decarboxylase	NRRL3_01409	AN10520	72%
β-ketoadipate:succinyl-CoA transferase	NRRL3_01886	AN10495^a^	86%
β-ketoadipyl-CoA thiolase	NRRL3_01526	AN5698	91%
Essential protein of unknown function	NRRL3_00837	AN5232^a^^b^	72%

a Proteins previously assigned by Martins *et al*. (30).

b Protein previously assigned by Martins *et al*. (30) as 3-carboxymuconolactone hydrolase/decarboxylase.

Previous studies used transcriptome analysis, 3,4-DHB degradation by strains overexpressing *NRRL3_01405*, purified enzyme activity, and mutant growth phenotype to conclude that this gene encodes protocatechuate 3,4-dioxygenase in *A. niger* (31, 32). Furthermore, our study showed the purified enzyme produced 3-carboxymuconic acid from 3,4-DHB, and the Δ*prcA* strain accumulated 3,4-DHB on a medium containing quinic acid as a carbon source. Residual growth in this strain may be due to the ability of one of the other catechol oxygenases encoded in the genome to catalyze this step, although in a previous study we were unable to detect activity of purified forms of these enzymes, 2 hydroxyquinol dioxygenases (NRRL3_02644 and NRRL3_05330) and a catechol dioxygenase (NRRL3_04787), on 3,4-DHB (32). However, Lubbers *et al*. (31) showed that a strain with deletions of both *prcA* and the hydroxyquinol dioxygenase, encoded by *NRRL3_02644*, converted 3,4-DHB slowly to hydroxyquinol, suggesting the possible existence of an enzyme that catalyzes the oxidative decarboxylation of 3,4-DHB to hydroxyquinol, as in the fungus *Trichospon cutaneum* (48). This enzyme has been suggested to be encoded by *NRRL_04986* (37).

Only one gene, *NRRL3_02586* (*cmcA*), displays strong homology to a known *N. crassa* orthologue with 3-carboxy-*cis,cis*-muconate cyclase activity (40). The corresponding *A. nidulans* orthologue, AN1151 (Table 3), was upregulated in both the transcriptome and proteome of benzoate-grown cells and a knockout of this gene eliminated growth on benzoate and resulted in the accumulation of an intermediate putatively identified as 3-carboxymuconate (30).

Transcriptome analysis showed that *cmcA* was upregulated 60-fold when *A. niger* was cultured with 3,4-DHB, compared with fructose, as the sole carbon source (Table 1). In Δ*cmcA*, the complete lack of growth (Fig. 2) establishes that it is required for growth on 3,4-DHB. Our biochemical analysis establishes that CmcA catalyzes the conversion of 3-carboxy-*cis,cis*-muconic acid to the 3-carboxymuconolactone that has been reported in other fungal systems, as opposed to the bacterial 4-carboxymuconolactone (19). As expected, the Δ*cmcA* strain accumulated a compound with the mass of 3-carboxy-*cis,cis-muconic acid* in the medium (Fig. 3*D*). Although there were several other compounds with a similar score, the Agilent Molecular Structure Correlator software predicted 3-carboxy-*cis,cis*-muconic acid as one of the most likely compounds based on the MS/MS fragment spectra.

Four candidates for the catabolism of 3-carboxymuconolactone were identified, of which *NRRL3_08340* was at least 3.5 times more highly induced than the other candidates (Table 1). The Δ*NRRL3_08340* strain, however, grew unimpeded on 3,4-DHB indicating that it is not essential for the pathway. This should perhaps not be surprising since sequence comparisons suggest that it is an orthologue of a bacterial enzyme involved in the decarboxylation of 4-carboxymuconolactone, rather than the 3-carboxymuconolactone produced by fungi, including *A. niger* as we have shown here. Deletion of each of the remaining two 3,4-DHB-induced candidates resulted in no growth on 3,4-DHB in Δ*NRRL3_00837* strain and severely reduced growth in the Δ*NRRL3_01409* strain, possibly due to activity of an alternative enzyme. No pathway-related metabolites accumulated in either deletion strain. However, biochemical analysis showed unambiguously that purified NRRL3_01409 catalyzed the conversion of 3-carboxymuconolactone to β-ketoadipate, whereas purified NRRL3_00837 did not. These results show the importance of using multiple different approaches when making gene assignments. Since deletion of the *A. nidulans* orthologue (*AN5232*) of *NRRL3_00837* resulted in a lack of growth and the appearance of a small amount of unconfirmed intermediate from benzoate (30), we tested the possibility that it had some kind of accessory role in the conversion. However, the addition of NRRL3_00837 has no effect on either the rate or nature of products from NRRL3_01409 (Fig. 5).

The protein encoded by NRRL3_01409 is consistent with the size of an *A. niger* enzyme that was purified previously and shown to possess both decarboxylase and hydrolase activities (33). A BLASTP comparison shows that the sequence over the C-terminal half of the protein is 28% identical with a 3-oxoadipate enol-lactonase from the bacterial protocatechuate branch of the β-ketoadipate pathway in *A. baylyii* (40). Furthermore, an α/β hydrolase domain (pfam00561) was detected in the C-terminal. The N-terminal half contains an uncharacterized protein domain (IPR003497, BRO N-terminal domain). Together with the biochemical characterization, these observations suggest two domains in this bifunctional enzyme: a hydrolase domain at the C-terminal and a decarboxylase domain at the N-terminal. The observation that the Δ*NRRL3_01409* strain still shows weak growth on 3,4-DHB suggests the possibility that paralogous enzymes, potentially those involved in the catechol branch of the β-ketoadipate pathway may be partly functional in the delactonization step. Motif analysis of NRRL3_00837 based on InterProScan shows the presence of the AhpD domain (InterPro entry IPR029032). However, a BLASTP comparison (using default parameters) between the sequence of NRRL3_00837 and AhpD, a characterized alkylperoxide reductase from *M. tuberculosis* (AHPD_MYCTU) upon which the domain is predicted, revealed no significant sequence identity.

To gain additional insight into the function of NRRL3_01409 and NRRL3_00837, we used AlphaFold (49) to predict their structures and to search for proteins that are structurally similar to them. Using the AlphaFold model of NRRL3_01409 as a query, the best hit was to crystal structure 2XUA from *Paraburkholderia xenovorans* in the Protein Data Bank (https://www.rcsb.org). Crystal structure 2XUA is described as a β-ketoadipate enol-lactonase because it displays 38% identity to the biochemically characterized *A. baylyi* β-ketoadipate enol-lactonase. Crystal structure 2XUA matches closely the AlphaFold-predicted structure of *A. baylyi* β-ketoadipate enol-lactonase (Fig. S12) and to the C-terminal half of NRRL3_01409 (Fig. S13). These results support our conclusion that the C-terminal half of NRRL3_01409 possesses hydrolase activity similar to that of β-ketoadipate enol-lactonase and that the N-terminal half of NRRL3_01409 is a previously uncharacterized structure with decarboxylase activity. The AlphaFold model of NRRL3_00837 showed no similarity to the AlphaFold or crystal structures of fungal or bacterial enzymes involved in 3,4-DHB catabolism except to the *A. nidulans* ortholog AN5232. A structural similarity search of the Protein Data Bank using the AlphaFold predicted model of NRRL3_00837 returned a transcription regulator, FapR, from *Staphylococcus aureus* (PDB ID 4A0Z). This may suggest NRRL3_00837 plays a role in the regulation of the β-ketoadipate pathway, though no significant similarity was found in the sequences of these two proteins.

For the last two steps of the pathway, conversion of β-ketoadipate to acetyl-CoA and succinate *via* β-ketoadipyl-CoA, three candidates each were identified based on homology, of which *NRRL3_01886* (encoding β-ketoadipate:succinyl-CoA transferase, KstA) and *NRRL3_01526* (encoding β-ketoadipyl-CoA thiolase, KctA) were each 70-fold upregulated by 3,4-DHB. Deletion of each gene in turn resulted in near-complete or complete loss of growth on 3,4-DHB, as well as on 2,3-DHB, which is likely degraded *via* β-ketoadipate. But as catabolism of 2,3-DHB does not use the enzymes between 3,4-DHB and β-ketoadipate, deletion of their encoding genes had no effect on the growth of 2,3-DHB (Fig. 2).

As would be expected from its predicted function as β-ketoadipate CoA transferase, Δ*NRRL3_01886* accumulated β-ketoadipate from quinic acid. Unfortunately, no metabolites were detected in culture supernatants for Δ*NRRL3_01526*, possibly since its expected product, β-ketoadipyl-CoA, would not be expected to pass the cell membrane. However, attempted analysis of intracellular metabolites did not reveal accumulation either. Although we attempted to heterologously express *NRRL3_01886*, only inclusion bodies were produced, so we were unable to confirm this functional assignment by direct biochemical analysis. Interestingly, it has been shown in bacteria such as *Pseudomonas putida* that β-ketoadipate CoA transferase is a heteromeric enzyme encoded by two genes (27). These authors noted the similarity of each gene to the two halves of homodimeric pig heart succinyl-CoA:3-ketoacid-CoA transferase and concluded that a gene fusion event occurred during evolution of the eukaryotic enzyme. As noted in the NRRL3 annotation for this gene, the closest orthologue (56% identity) in the Uniprot database to KstA is human succinyl-CoA:3-ketoacid coenzyme A transferase (accession number P55809), with an amino-terminal corresponding to one subunit (InterPro domain IPR012792) and the C-terminal encompassing the other subunit (InterPro domain IPR012791).

## Experimental procedures

### Chemicals

The chemicals used were generally the purest available. For use as growth substrates, fructose, glucose, sucrose, quinic acid; 2,3-dihydroxybenzoic acid, and 3,4-dihydroxybenzoic acid were purchased from Sigma-Aldrich (Oakville, ON).

### Strains and growth conditions

The *A. niger* strains used and constructed in this study are listed in Table 4. The parental strain used for mutational analysis was CBS 138852, derived from strain N593 (NRRL3 (N400) → N402 → N593 (ATCC 64973)), which is auxotrophic for uridine with short conidiophores and carries a deletion of the *kusA* gene to increase the efficiency of homologous recombination (50). Transformants were grown for 5 days at 30 °C on selective minimal media plates (51). For extraction of genomic DNA for screening by PCR, spores from transformants were inoculated into 250 μl of complete media (minimal media supplemented with 5 g/L yeast extract, 1 g/L casamino acids, and 10 mM uridine) and grown at 30 °C for 17 h. Phenotypic testing was performed by spotting 2 μl of saline/Tween containing 1000 fresh spores onto minimal media plates with 0.5% fructose or various aromatic compounds as the sole carbon source. The DH5α strain of *E. coli* was used for the maintenance and propagation of cloned plasmids. For mass spectrometric analysis of metabolites, 200 ml of complete media was inoculated with fresh spores to a concentration of 2 × 10^6^ spores/ml and grown overnight at 30 °C with shaking at 220 rpm. Mycelia (250 mg) were transferred to 25 ml of minimal media with 0.5% quinic acid as the sole carbon source and grown at 30 °C for up to 48 h, shaking at 220 rpm.

**Table 4:** *Aspergillus niger* strains used in this study

Strain	Genotype	Source
N402	N400 (NRRL3) *cspA*, *cspB*	(49, 67)
CBS 138852	N593 (ATCC 64973) Δ*kusA*	(50)
MS4	CBS 138852 Δ*prcA* (*NRRL3_1405*)	This study
MS5	CBS 138852 Δ*cmcA* (*NRRL3_2586*)	This study
MS6	CBS 138852 Δ*NRRL3_00837*	This study
MS7	CBS 138852 Δ*chdA* (*NRRL3_01409*)	This study
MS8	CBS 138852 Δ*NRRL3_08340*	This study
MS9	CBS 138852 Δ*kstA (NRRL3_01886)*	This study
MS10	CBS 138852 Δ*kctA (NRRL3_01526)*	This study

Two cultivation methods of *A. niger* strain N402 were used to prepare RNA for transcriptome analysis; shake flask and bioreactor batch fermentations. For shake flash fermentation, spores at 2 × 10^6^/ml were added to complete media with 0.75% fructose as carbon source and grown at 30 °C, shaking at 220 rpm. Following overnight cultivation, mycelia were washed with minimal media lacking a carbon source, and 250 mg of mycelia were transferred into 25 ml minimal media containing either 0.75% fructose or 0.75% 3,4-DHB as the sole carbon source and grown at 30 °C, shaking at 220 rpm. Two hours after transfer to fresh media, mycelia were harvested for RNA extraction. Bioreactor-controlled batch fermentations on 3,4-DHB were performed as described previously (52, 53). In short, autoclaved bioreactor vessels were filled with 5 L of sterile MM containing 0.75% 3,4-DHB as a carbon source. During cultivation at 30 °C, the controller was set to maintain pH 3 by the addition of titrants (2 M NaOH or 1 M HCl). Sterile air was supplied at a rate of 1 L min^−1^. Prior to inoculation, 1.5 ml of 10% (w/v) filter-sterilized yeast extract was added to enhance conidial germination. Cultures were inoculated with freshly harvested spores at a concentration of 7.0 × 10^8^ conidia per liter. To reduce the loss of hydrophobic conidia during germination, the stirrer speed was set to 250 rpm and the culture was aerated *via* the headspace during the first 6 h after inoculation. Subsequently, the stirrer speed was increased to 750 rpm, 0.5 ml of polypropyleneglycol P2000 was added as an antifoam agent, and air was supplied *via* the sparger. Cultures broth was harvested at regular intervals from batch cultures and mycelial biomass was retained by vacuum filtration using glass microfiber filters (Whatman, Maidstone, UK). Both biomass and filtrate were quickly frozen in liquid nitrogen and subsequently stored at −80 °C. Dry biomass concentrations were gravimetrically determined from lyophilized mycelia originating from a known mass of culture broth. RNA was isolated from mycelium that was grown until the mid-exponential phase.

### RNA sequencing and transcriptome analysis

RNA-seq data from the transfer culture samples of *A. niger* strain N402 grown on 3,4-DHB and fructose, as well as the bioreactor samples grown on 3,4-DHB, were deposited in the Sequence Read Archive under accession number SRP410706. RNA-seq data regarding *A. niger* grown on fructose which was used as a reference for the bioreactor samples was previously deposited in the Sequence Read Archive under accession number SRP078485 (53).

Sequencing was performed at the Centre d’expertise et de services Génome Québec using Illumina HiSeq 4000 paired-end sequencing technology with a read length of 100. The raw RNA-seq reads were pre-processed with the bbduk.sh script in the BBMap package (54) to trim sequencing adapters and remove reads derived from phiX and ribosomal RNA. Pre-processed RNA-Seq data were aligned to the NRRL3 genome using the RSubread software (55). Differential expression analysis was performed using R (https://www.R-project.org, (56)), Rstudio (http://www.rstudio.com, (57)), and the DESeq2 package (58). Experimental conditions were compared to the fructose control with a false discovery rate of 10%.

### Mutant generation using CRISPR/Cas9

CRISPR/Cas9 was used to generate mutants by creating deletions in target genes as previously described (59). CRISPR/Cas9 guide RNA sequences were identified using Geneious R9.1 (http://www.geneious.com, (60)). Guide RNA expression cassettes were inserted into the plasmid ANEp8-Cas9 using ligation-independent cloning as described (59) (Fig. S1). Sequences chosen as the CRISPR target sequences are listed in Figs. S2–S8. Single-stranded 60-nucleotide gene-editing oligonucleotides with 30 bases of homology on each side of the targeted deletion region were introduced into *A. niger* along with the CRISPR plasmids by co-transformation. These oligonucleotides were used to repair the chromosomal breaks cleaved by the Cas9 nuclease to create deletion mutants, as shown in Figs. S2–S8.

### *A. niger* gene transformation and mutations verification

*A. niger* protoplasts were generated using young hyphae as previously described (61). Transformations were performed as previously described (62) using 1.5 μg of CRISPR plasmid DNA and 1 nmol of the corresponding gene-editing oligonucleotide (Figs. S2–S8). Two or more independent transformants for each gene were isolated for phenotypic analysis. Mutations in the transformants were verified by PCR amplification of sequences surrounding the deletion site using primers listed in the descriptions of Figs. S2–S8. Amplified DNA fragments were visualized on agarose gel (Figs. S2–S8).

## Analysis of metabolites accumulated in deletion mutants by liquid chromatography-mass spectrometry

Following the transfer of mycelia from triplicate cultures of parental and mutant strains into minimal media containing quinic acid, samples of the cultures were collected at various time points in 1.5 ml microcentrifuge tubes. Samples were centrifuged at 13,300 rpm for 30 min to remove mycelia. Supernatants were transferred to new tubes and an equal volume of −20 °C methanol was added. Samples were incubated on ice for 10 min and centrifuged at 13,300 rpm for 30 min to precipitate proteins. Supernatants were transferred to new tubes and an equal volume of 0.1% formic acid was added.

Electrospray liquid chromatography-mass spectrometry was performed on an Agilent 6560 Ion mobility Q-TOF and 1290 Infinity II LC/MS system (Agilent Technologies, Santa Clara, CA). The scan range was from 100 to 1400 m/z. Reversed-phase liquid chromatography was performed using a Synergy 4 μm Hydro-RP 80 Å, 150 × 2.00 mm column (Phenomenex, Torrance, CA). Analysis was performed using Agilent MassHunter workstation software. Concentrations of compounds were calculated by performing LC/MS using standards of known concentration to generate a graph of the peak area of the extracted ion chromatogram of the compound vs. concentration. A trendline calculated based on these results was used to calculate the concentration in the culture samples.

### Recombinant production of candidate enzymes and purification

Genes encoding candidate enzymes were PCR amplified using Phusion High-Fidelity DNA Polymerase (New England Biolabs) with cDNA isolated from alfalfa-barley grown *A. niger* as a template. Table S1 lists the primers used for amplifying the target cDNA.

After PCR amplification using a standard or Touchdown (63) protocol, correctly-sized products were excised from a 1% agarose gel and purified using the Roche High Pure PCR Product Purification Kit (Roche, Basel, Switzerland) before insertion into the pLATE11 (*NRRL3_01409* and *NRRL3_02586*) or pLATE52 (*NRRL3_00837*) vectors by ligation-independent cloning method (aLICator LIC cloning kit (Thermo Scientific, Waltham, MA)). Plasmids were introduced by gene transformation of *E. coli* DH5 by mixing with chemically competent cells, heat shock, and plating on LB agar containing carbenecillin (50 μg/ml) (64). Single colonies were picked after overnight growth and plasmids were purified using the BioBasic EZ-10 Spin Column Plasmid DNA Minipreps Kit. Plasmids were screened using PCR amplification, and those with correctly-sized inserts were further subjected to DNA sequencing to confirm the identity of the inserted gene.

For protein production, plasmids were introduced by gene transformation into *E. coli* BL21(DE3), grown on LB in the presence of either carbenecillin (50 μg/ml) or ampicillin (100 μg/ml) before induction with IPTG (0.4 mM) in mid-exponential phase, then incubated with shaking at 16 °C and 225 rpm for 16 h. Cells were harvested by centrifugation at 7500*g* at 4 °C for 20 min, and the pellet was resuspended in 50 mM Tris-HCl buffer, pH 7.5 or 50 mM HEPES, pH 8.6, containing DTT 0.2 mM. Cell suspensions on ice were sonicated using a BioLogics Model 300 VT ultrasonic homogenizer (BioLogics, Manassas, VA) at 50% full power for 10 bursts of 10 s each. This was followed by centrifugation at 6000*g* for 1 h. The supernatant (“crude extract”) was used to purify the enzymes of interest.

A Fast Flow DEAE-Sepharose column (Sigma-Aldrich, 2.6 cm diameter × 27.5 cm) was used for the purification of NRRL3_01409 and NRRL3_02586. The column was equilibrated with 50 mM Tris-HCl pH 7.5 before the application of crude extracts. The column was washed with 48 ml of 50 mM Tris-HCl pH 7.5 and the bound proteins were eluted with an 888-mL gradient of 0 to 1 M NaCl in 50 mM Tris-HCl pH 7.5. Fraction purity was examined by SDS-PAGE, and the fractions with the highest concentration of the target protein were combined and concentrated using an Amicon concentrator (Sigma-Aldrich) with a 10 kDa cutoff membrane. The concentrated preparation was loaded onto a Sephacryl S-300 column (Sigma-Aldrich, 2.6 cm diameter × 76 cm) equilibrated with 50 mM Tris-HCl pH 7.5 and eluted with 840 ml of the same buffer at 3 ml/min. Fractions with the highest purity of the target protein based on SDS-PAGE examination were combined, concentrated, equilibrated to 20% ammonium sulfate with 50 mM Tris-HCl pH 7.5, and loaded onto a phenyl-Sepharose column (2.6 cm × 11.8 cm) equilibrated with 20% ammonium sulfate in 50 mM Tris-HCl pH 7.5. The column was washed with 2 column volumes of 50 mM Tris-HCl pH 7.5 followed by gradient elution over 650 ml from 20% to 0% ammonium sulfate in 50 mM Tris-HCl pH 7.5. Fraction purity was examined by SDS-PAGE, the fractions with the highest concentration of the target protein were combined, concentrated and buffer exchanged with 50 mM Tris-Cl pH 7.5 using an Amicon concentrator with a 10 kDa cutoff membrane, then stored at −80 °C.

For purification of the recombinant NRRL3_01405 and NRRL3_00837, the crude extract was loaded at 1 ml/min onto a His-trap column (5 ml) equilibrated with 50 mM Tris-HCl pH 7.5, 0.1 M NaCl and 5 mM imidazole, and 5 ml fractions were collected. Unbound proteins were eluted using 10 column volumes of 50 mM Tris-HCl pH 7.5, 0.1 M NaCl, and 5 mM imidazole, then a step-wise elution using 50 mM Tris-HCl pH 7.5, 0.1 M NaCl and eluted with imidazole concentrations of 20 mM, 200 mM and 500 mM. Fraction purity was examined by SDS-PAGE, those containing pure NRRL3_00837 were combined and concentrated using an Amicon concentrator with a 10 kDa cutoff membrane, then diluted successively with two 5-fold volumes of 50 mM Tris-Cl pH 7.5 containing 0.1 M NaCl. The purified protein was aliquoted and stored at −20 °C or −80 °C with 20% glycerol added.

The identity of the purified recombinant proteins was verified by peptide mass fingerprinting. Briefly, liquid chromatography-tandem MS (LC-MS/MS) analyses were performed on a Thermo EASY nLC II LC system coupled to a Thermo LTQ Orbitrap Velos mass spectrometer equipped with a nanospray ion source. Proteins were in-gel digested using trypsin for 16 h at 3%. A volume of 2 μl of each sample containing around 100 ng of tryptic peptides was injected onto a 10 cm × 100 μm column in-house packed with Michrom Magic C18 stationary phase (5 μm particle diameter and 300 Å pore size). Peptides were eluted using a 35-min gradient at a flow rate of 400 nl/min with mobile phase A (96.9% water, 3% ACN, and 0.1% FA) and B (97% ACN, 2.9% water, and 0.1% FA). A full MS spectrum (*m/z* 400–1400) was acquired in the Orbitrap at a resolution of 60,000, then the 10 most abundant multiple-charged ions were selected for MS/MS sequencing in a linear trap with the option of dynamic exclusion. Peptide fragmentation was performed using a collision-induced dissociation at a normalized collision energy of 35% with an activation time of 10 ms. The MS data were processed using Thermo Proteome Discoverer software 2.4 (RRID:SCR_014477) with the SEQUEST search engine. Database searches were against the sequences of the target proteins, the UniProt *E. coli* proteome database (Uniprot UP000002032), and cRAP protein sequences (https://www.thegpm.org/crap/). Peptide coverage observed was 78.1% for NRRL3_01405, 86.9% for NRRL3_02568; 92.5% for, NRRL3_01409; and 91.9% for NRRL3_00837.

### Enzyme activity

The activities of protocatechuate dioxygenase, β-carboxymuconate lactonizing enzyme, and β-carboxymuconolactone decarboxylase/hydrolase were measured at room temperature by the changes over time in absorbance at 290 nm, 260 nm, and 230 nm, respectively, as described previously (28). Substrates were generated *in situ* using purified enzymes starting from 3,4-dihydroxybenzoic acid (3,4-DHB). Each substrate was prepared in triplicate in volumes of 10 ml and three separate readings were recorded for each enzyme.

### Identification of intermediates generated *in vitro*

(a) UV spectroscopy. Conversion of 3,4-DHB (100 μM in 1 ml 100 mM Tris-HCl pH 7.5) was followed by scanning the UV spectrum from 300 to 220 nm using a Cary 50 spectrometer at a scan rate of 360 nm/min, after the successive addition of various enzymes. A blank containing only enzyme was subtracted for each reaction mixture.
(b) Isolation of β-carboxymuconolactone and β-ketoadipate. In a 125 ml Erlenmeyer flask, 9.25 mg of 3,4-DHB was dissolved in 20 ml of 50 mM Tris-HCl pH 7.5 containing 60 μg of NRRL3_01405 (PrcA, protocatechuate 3,4-dioxygenase) and 515 μg of NRRL3_02586 (CmcA, 3-carboxy-*cis,cis*-muconate cyclase) to produce β-carboxymuconolactone at room temperature on a rotary shaker. β-ketoadipate was produced under the same conditions with the addition of 150 μg of NRRL3_01409 (ChdA, 3-carboxymuconolactone hydrolase/decarboxylase). An aliquot was withdrawn and a UV spectrum from 220 to 300 nm was taken every 30 min until no further spectral changes were observed.

Following the accumulation of β-ketoadipate or β-carboxymuconolactone, NaCl (2 g) was added and dissolved, followed by acidification with 12 drops of 6 M HCl. The solution was then transferred in 5 ml-aliquots into 20 ml vials. An equal part of ethyl acetate was added to each aliquot and vortexed over 2 min. Once the phases were separated, the organic layer was transferred into a 30 ml vial. This extraction process was repeated twice for each aliquot and then frozen at −80 °C for 10 min. The extract was then thawed at room temperature for 10 min and the remaining aqueous layer was removed from the bottom of the vial. The solvent was evaporated with a stream of air and the residue was stored at −80 °C.

(c) ^1^H NMR. All experiments were run on a Varian VNMRS-500 MHz equipped with 5 mm AutoX DB (Dual Broadband) probe^1^H-^19^ F/X[^15^N-^31^P], with z-PFG and automatic tuning for all nuclei by the ProTune accessory. The system operates with VNMRJ 3.2 software under LINUX Red Hat 5.
(d) Mass Spectrometry of *in vitro* generated compounds. Compounds generated from reactions with purified proteins *in vitro* were run on a Q-ToF3 (Waters Micromass, Milford, MA) equipped with electrospray ionization. The samples were injected directly into the spectrometer. Analysis was performed using the MassLynx NT software. NMR spectra were referenced to tetramethylsilane (TMS).

### Protein modeling

NRRL3 protein 3-day models were generated using Alphafold (65) version 2.3.0 in reduced mode with a max template date of 2023 to 05 to 01. Predicted structures were compared to similar proteins available from the Protein Data Bank (experimentally determined) or UniProt (predicted) using the ChimeraX (66) modeling software.

### Gene accession numbers

The *A. niger* NRRL3 genes examined in this study are available in the GenBank database under the following accession numbers: OR137151 (*NRRL3_01405*), OR137152 (*NRRL3_02586*), OR137153 (*NRRL3_01409*), OR137154 (*NRRL3_00837*), OR137155 (*NRRL3_08340*), OR137156 (*NRRL3_01886*), and OR137157 (*NRRL3_01526*).

## Data availability

All data are contained within the manuscript.

## Supporting information

This article contains supporting information (58, 59).

## Conflict of interest

The authors declare that they have no conflicts of interest with the contents of this article.
