# Supplementary material for: Functional analysis of the protocatechuate branch of the β-ketoadipate pathway in Aspergillus niger
Source: J Biol Chem. 2023 Jul 1;299(8):105003. doi: 10.1016/j.jbc.2023.105003 (PMC10406623; doi:10.1016/j.jbc.2023.105003)
Supplement: Supporting information [file mmc1.pdf]

Functional analysis of the protocatechuate branch of the β-ketoadipate pathway in *Aspergillus niger*

Michael Sgro[1,2], Nicholas Chow[3], Farnaz Olyaie[3], Mark Arentshorst[4], Nicholas Geoffrion[2], Arthur F.J. Ram[4], Justin Powlowski[2,3+] and Adrian Tsang*[1,2]

[1] Dept of Biology, Concordia University, 7141 Sherbrooke St. W., Montreal, Quebec H4B 1R6

[2] Centre for Structural and Functional Genomics, Concordia University, 7141 Sherbrooke St. W., Montreal, Quebec H4B 1R6

[3] Department of Chemistry and Biochemistry, Concordia University, 7141 Sherbrooke St. W., Montreal, Quebec H4B 1R6

[4] Institute of Biology Leiden, Microbial Sciences, Leiden University, Sylviusweg 72, 2333 BE Leiden, The Netherlands

[+] deceased

* For correspondence: Adrian Tsang, adrian.tsang@concordia.ca

Table of contents

Table S1 …………………………………………………………………………………….. S-3
Table S2 …………………………………………………………………………………….. S-4

Figure S1 ……………………………………………………………………………………. S-5
Figure S2 ……………………………………………………………………………………. S-6
Figure S3 ……………………………………………………………………………………. S-7
Figure S4 ……………………………………………………………………………………. S-8
Figure S5 ……………………………………………………………………………………. S-9
Figure S6 ……………………………………………………………………………………. S-10
Figure S7 ……………………………………………………………………………………. S-11
Figure S8 ……………………………………………………………………………………. S-12
Figure S9 ……………………………………………………………………………………. S-13
Figure S10……………………………………………………………………………………S-14
Figure S11……………………………………………………………………………………S-15
Figure S12……………………………………………………………………………………S-16
Figure S13……………………………………………………………………………………S-17

Table S1. Primers and oligonucleotides used in this study for construction of *E. coli* expression plasmids. Sequences added for ligation-independent cloning are in bold.

Primer	Sequence
NRRL3_02586F	5'-**AGAAGGAGATATAACT**ATGAAGCATCATCTTATGGTCGGTACCTGG-3'
NRRL3_02586R	5'-**GGAGATGGGAAGTCA**TTAATCATACCAAATTGCATTCATACCGAATCCC-3'
NRRL3_01409F	5'-**AGAAGGAGATATAACT**ATGGCGACCCCCGGC-3'
NRRL3_01409R	5'-**GGAGATGGGAAGTCA**CTAGTGCAGGTACTCATTCACCACCG-3'
NRRL3_01886F	5'-**AGAAGGAGATATAACT**ATGTCGCGGCCTGTTCC-3'
NRRL3_01886R	5'-**GGAGATGGGAAGTCA**TTACAGCATAGGCTTCAGATCAGC-3'
NRRL3_00837F	**5'-GGTTGGGAATTGCAA**ATGAGTCTCCCCTACGCC-3'
NRRL3_00837R	**5'-GGAGATGGGAAGTCA**TCACTTCCTCTCAAGATCAGCC-3'

Table S2 RNA sequencing data from bioreactor culture for genes with homology to 3,4-DHB pathway genes. Mean values are the average of duplicates. Complete data and DESeq2 (58) analysis shown in Table S4. Predicted genes in bold. TPM, transcripts per million.

Enzyme	Predicted gene	Mean Fructose TPM	Mean 3,4-DHB TPM	Fold change (3,4-DHB vs. Fructose)
Protocatechuate 3,4-dioxygenease	***NRRL3_01405***	**3.91**	**4246.07**	**1085.59**
	NRRL3_02644	15.15	87.23	5.76
	NRRL3_04277	0.99	1.21	1.22
	NRRL3_04787	21.46	49.63	2.31
	NRRL3_05330	3.01	6.03	2.00
Carboxy-cis,cis-muconate cyclase	***NRRL3_02586***	**38.91**	**325.91**	**8.38**
3-carboxymuconolactone hydrolase/decarboxylase	*NRRL3_00837*	14.13	103.06	7.30
	NRRL3_01409	**13.20**	**233.16**	**17.66**
	NRRL3_03759	36.52	36.57	1.00
	NRRL3_08340	0.66	1168.43	1769.81
β-ketoadipate:succinyl-CoA transferase	*NRRL3_01593*	24.86	68.34	2.75
	NRRL3_01886	**62.89**	**709.02**	**11.27**
	NRRL3_11640	80.04	216.39	2.70
β-ketoadipyl-CoA thiolase	***NRRL3_01526***	**29.76**	**499.99**	**16.80**
	NRRL3_07786	127.83	172.33	1.35
	NRRL3_11162	71.82	247.94	3.45

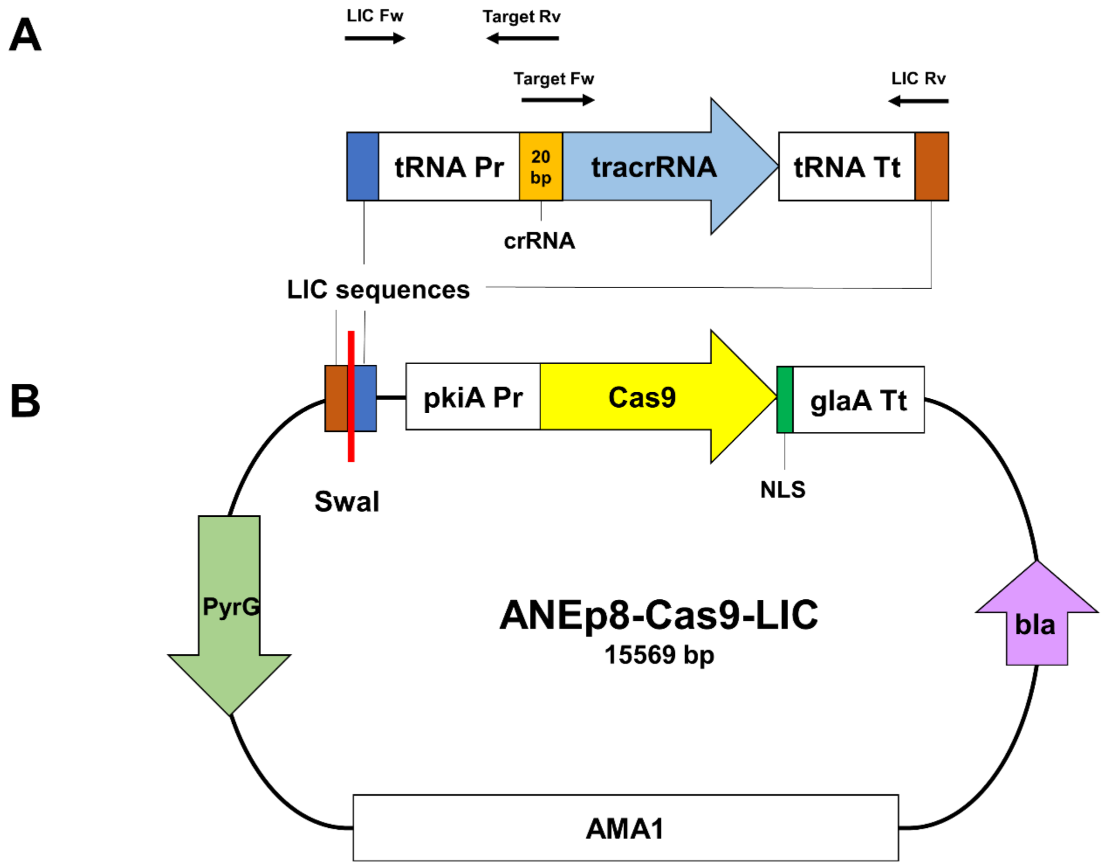

Figure S1. Diagram of CRISPR plasmid construction using ligation independent cloning. (A) Primers used to create the gRNA expression cassette by overlap PCR. The Target Fw and Target Rv primers, with sequence GTTTTAGAGCTAGAAATAGCAAG and GACGAGCTTACTCGTTTCG respectively, contained a unique 20 nucleotide tail for each target gene, which formed the gRNA to be expressed. The LIC Fw and LIC Rv primers had sequence **CAACCTCCAATCCAATTTG**ACTCCGCCGAACGTACTG and **ACTACTCTACCACTATTTG**AAAAGCAAAAAAGGAAGGTACAAAAAAGC respectively (LIC sequence in bold). (B) gRNA expression cassettes were inserted into the ANEp8-Cas9-LIC plasmid (59).

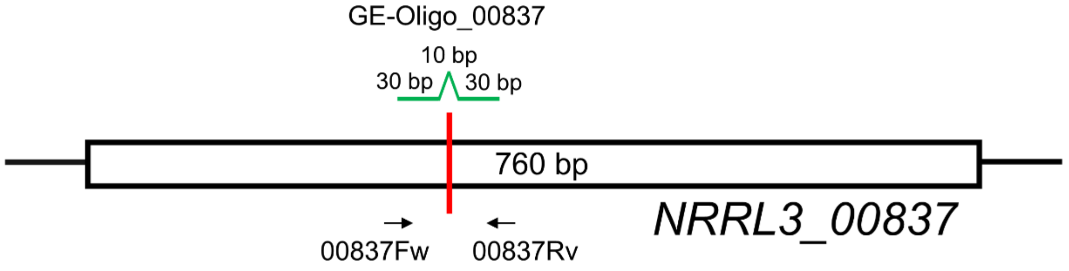

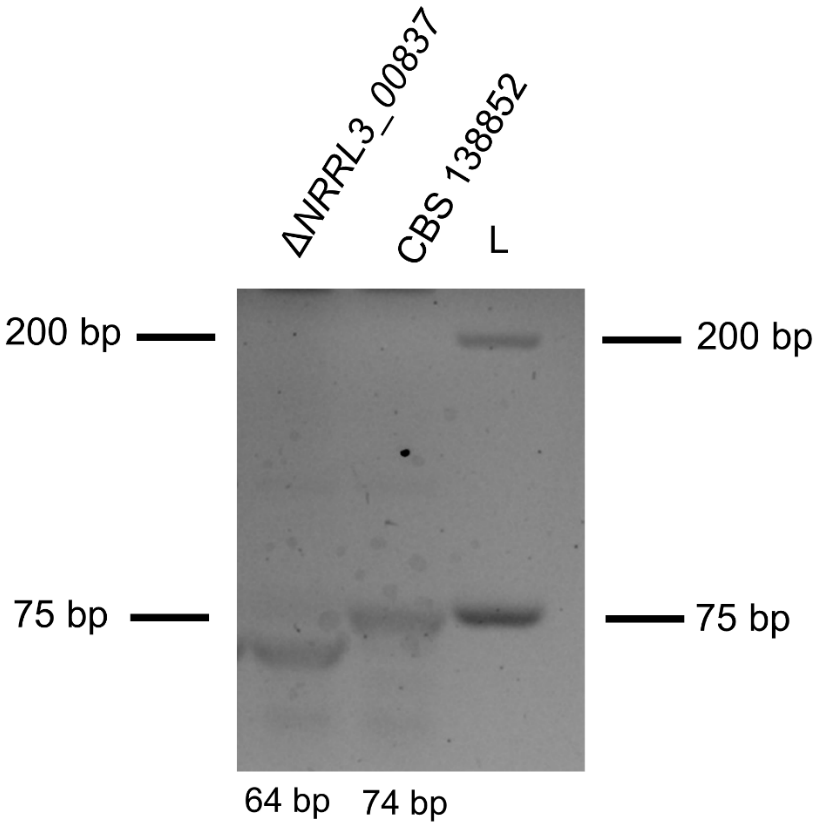

Figure S2. Diagram of mutant generation and verification method and gel used for verification of deletion in *NRRL3_00837*. CRISPR/Cas9 was used to create a double stranded break indicated by the red line using the target sequence ACGCAAACAATCATCGACGG. The gene-editing oligonucleotide GE-Oligo_00837 (green) with sequence TTCCTGGGCGCCATCCGCACGCAAACAATCGATTGCTCGAGCTCGCCGTGTGTCGCGTGG was used for homology directed repair to create a deletion of 10 bp. Primers around the deletion (00837Fw: AACAGCTTCCTGGGCGCCAT and 00837Rv: ACGCGACACACGGCGAG) were used for PCR verification. Expected band sizes are shown below each PCR product. GeneRuler 1 kb Plus DNA Ladder (Thermo Scientific) was run in lane L.

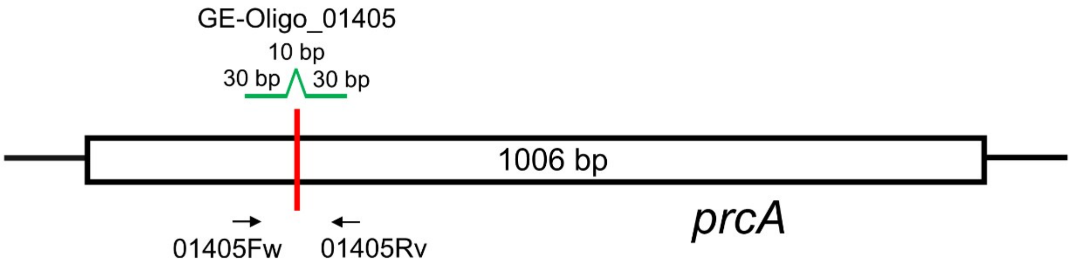

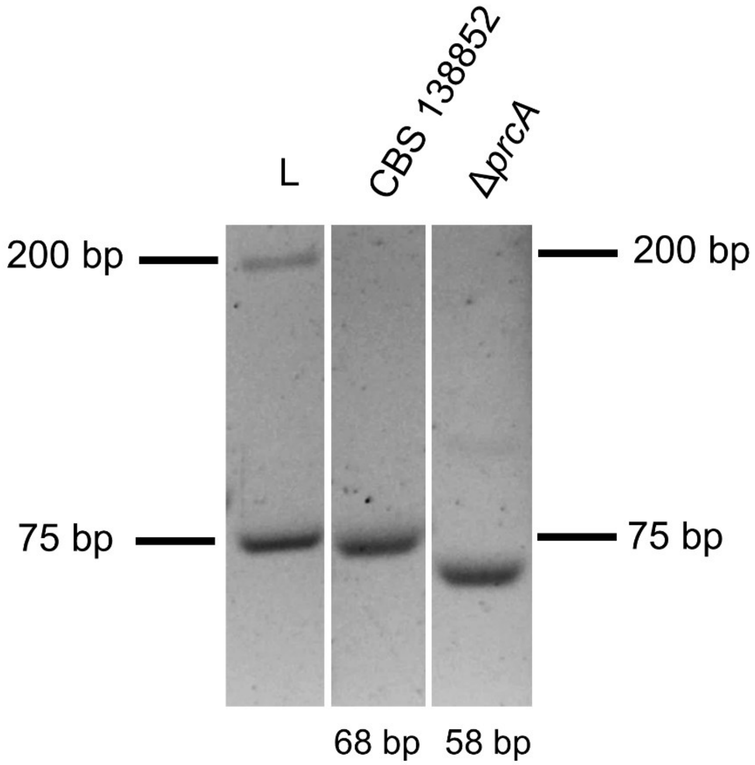

Figure S3. Diagram of mutant generation and verification method and gel used for verification of deletion in *prcA*. CRISPR/Cas9 was used to create a double stranded break indicated by the red line using the target sequence AGATACGGTGGCACTCGTTA. The gene-editing oligonucleotide GE-Oligo_01405 (green) with sequence AACTCCATTGGAAAGATCAGCACTCCCATCGCCACCGTATCTGCGATGTTATCGGTCTGG was used for homology directed repair to create a deletion of 10 bp. Primers around the deletion (01405Fw: AACTCCATTGGAAAGATCAG and 01405Rv: AGACCGATAACATCGCAG) were used for PCR verification. Expected band sizes are shown below each PCR product. GeneRuler 1 kb Plus DNA Ladder (Thermo Scientific) was run in lane L.

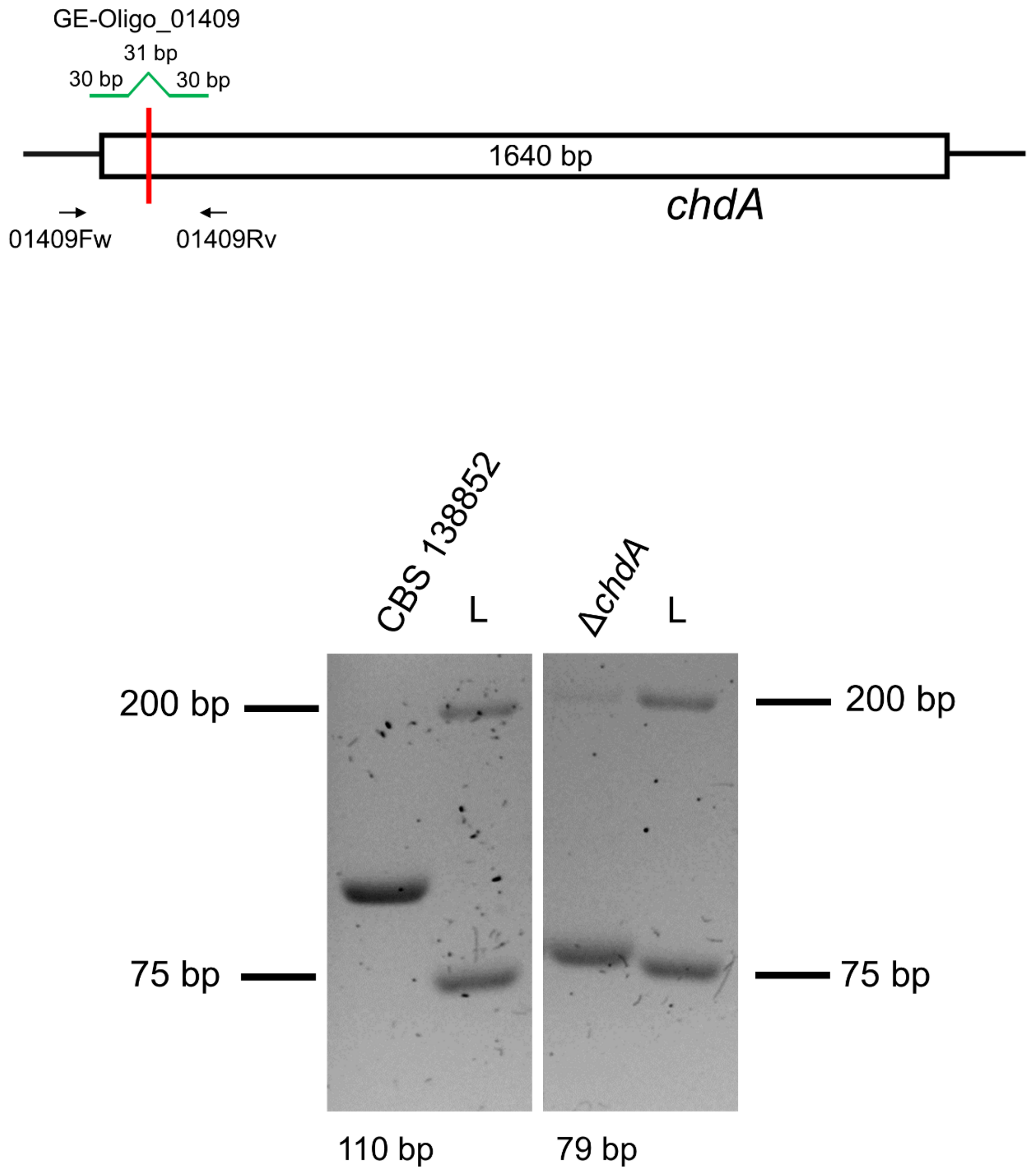

Figure S4. Diagram of mutant generation and verification method and gel used for verification of deletion in *chdA*. CRISPR/Cas9 was used to create a double stranded break indicated by the red line using the target sequence GTACCAGTCGTGGAATTGAG. The gene-editing oligonucleotide GE-Oligo_01409 (green) with sequence TTCTCTGGGTGACGATGAACCCTCGTCCCTCAACACCGAACACGGCCCTCTGCGCCTGCG was used for homology directed repair to create a deletion of 10 bp. Primers around the deletion (01409Fw: CGATGAACCCTCGTCCCT and 01409Rv: AGGGCCGTGTTCGGTGTT) were used for PCR verification. Expected band sizes are shown below each PCR product. GeneRuler 1 kb Plus DNA Ladder (Thermo Scientific) was run in lane L.

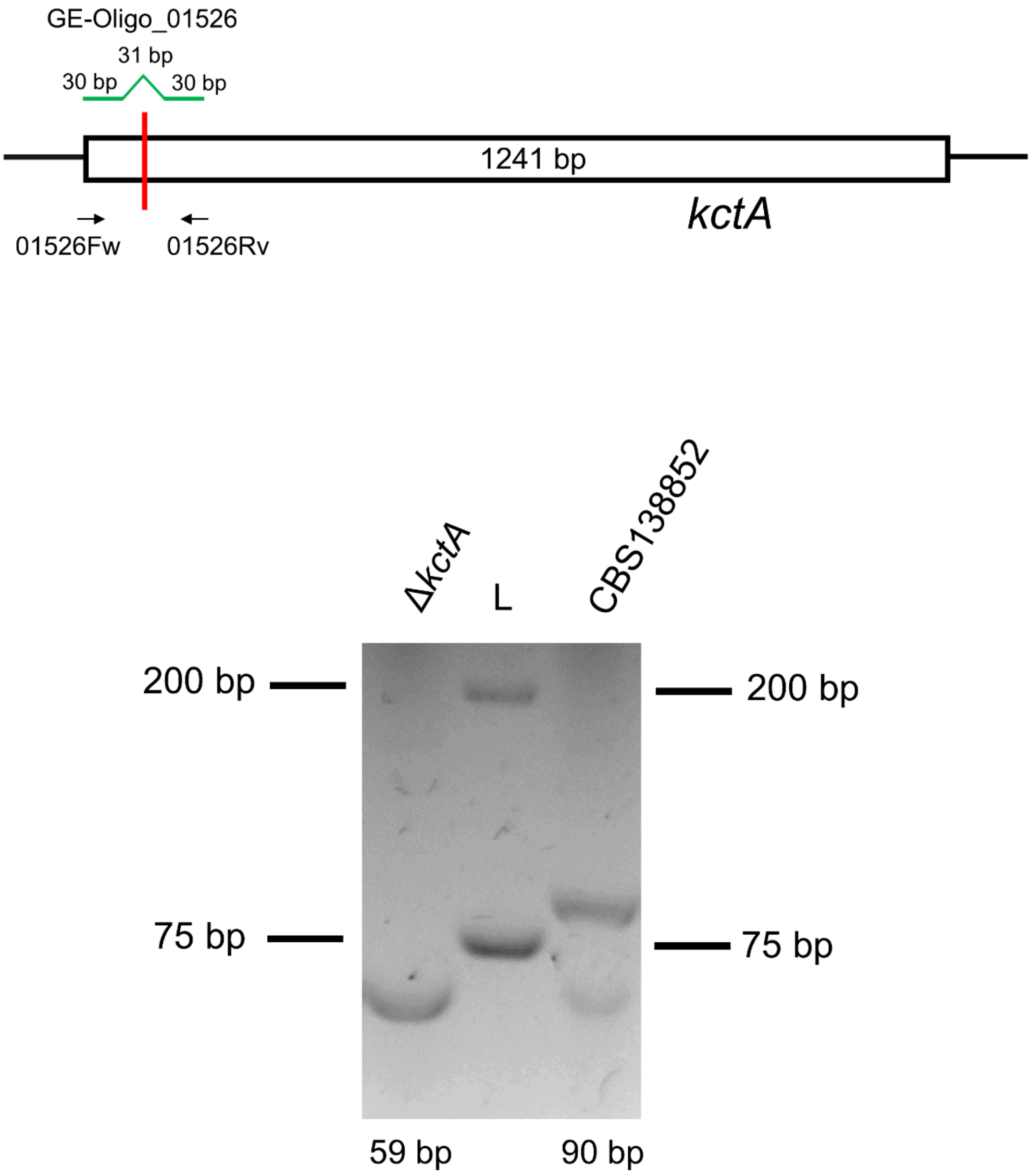

Figure S5. Diagram of mutant generation and verification method and gel used for verification of deletion in *kctA*. CRISPR/Cas9 was used to create a double stranded break indicated by the red line using the target sequence GGTCCTCCAGAAATCTCCCT. The gene-editing oligonucleotide GE-Oligo_01526 (green) with sequence CCTCTCCAATCCCCCGGGGCCTGCGCCAGGTCTCTCTTCCCTCCGTACCCCGGTCACCCG was used for homology directed repair to create a deletion of 10 bp. Primers around the deletion (01526Fw: GGCCTCTCCAATCCCC and 01526Rv: GGTACGGAGGGAAGAGAGAA) were used for PCR verification. Expected band sizes are shown below each PCR product. GeneRuler 1 kb Plus DNA Ladder (Thermo Scientific) was run in lane L.

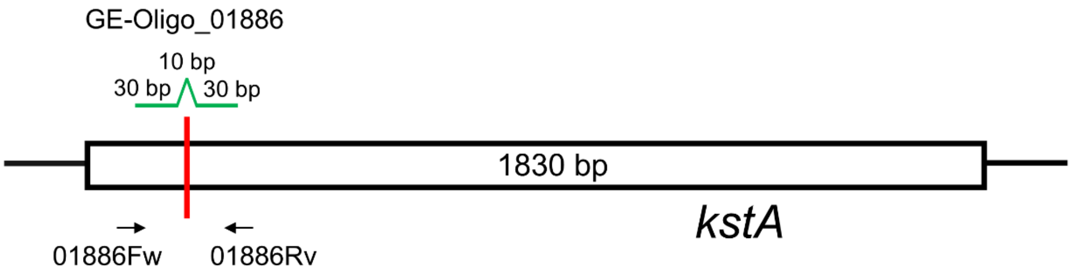

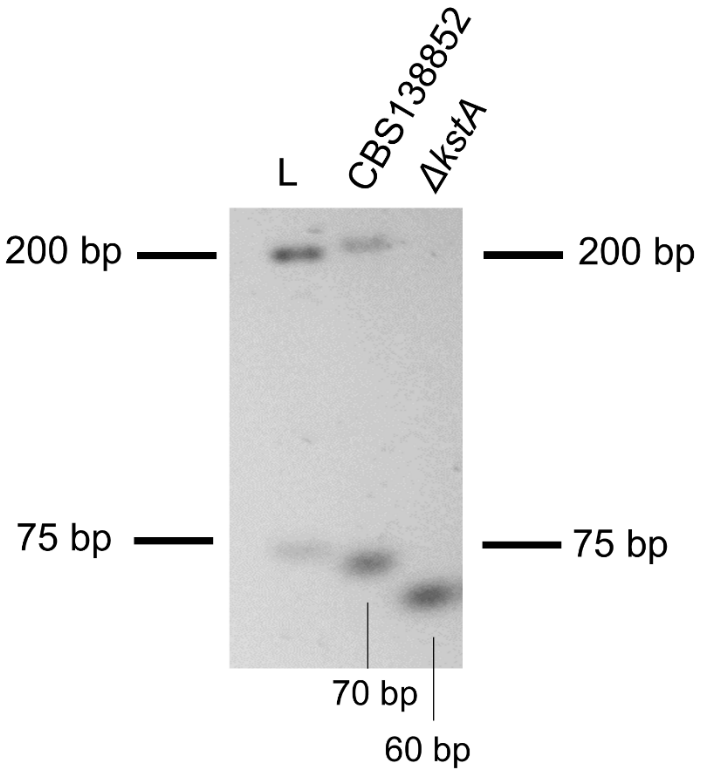

Figure S6. Diagram of mutant generation and verification method and gel used for verification of deletion in *kstA*. CRISPR/Cas9 was used to create a double stranded break indicated by the red line using the target sequence TCAGATCCTTGATGTCCGGG. The gene-editing oligonucleotide GE-Oligo_01886 (green) with sequence CCTACTACCTTGATTGATGCCGTGCGTGACTCAAGGATCTGACTGTCGTCTCCAACAATG was used for homology directed repair to create a deletion of 10 bp. Primers around the deletion (01886Fw: CCTACTACCTTGATTGATGC and 01886Rv: CATTGTTGGAGACGACAGTC) were used for PCR verification. Expected band sizes are shown below each PCR product. GeneRuler 1 kb Plus DNA Ladder (Thermo Scientific) was run in lane L.

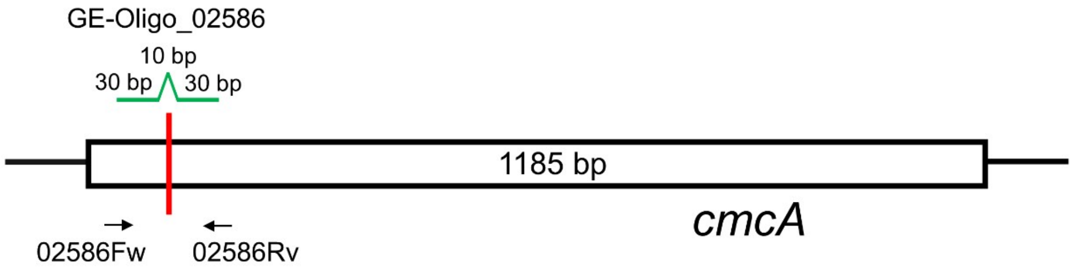

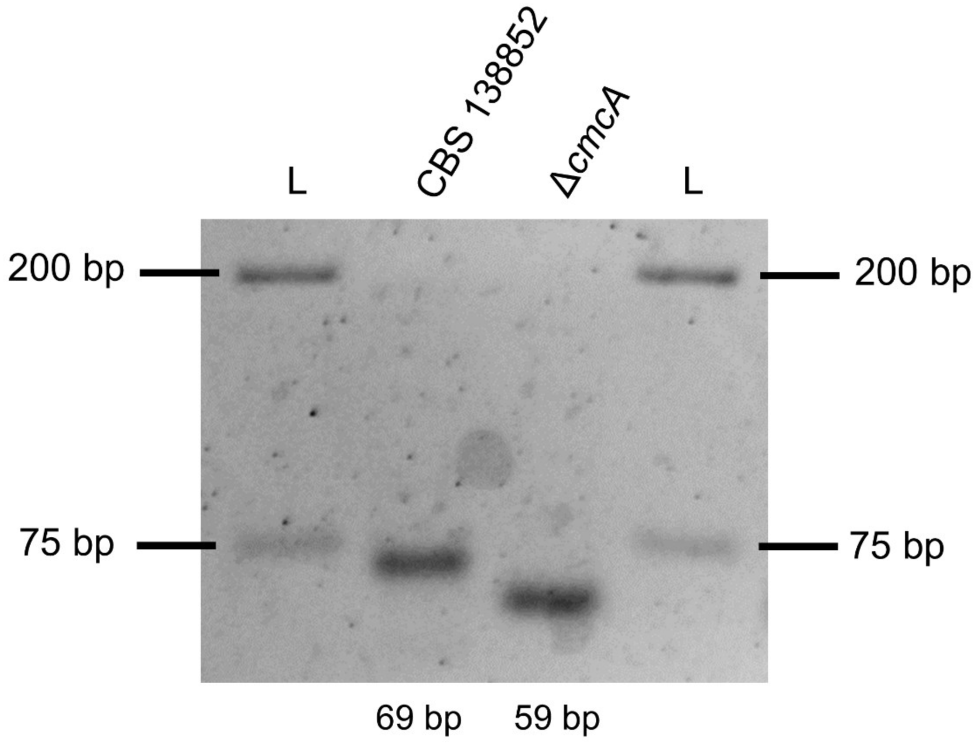

Figure S7. Diagram of mutant generation and verification method and gel used for verification of deletion in *cmcA*. CRISPR/Cas9 was used to create a double stranded break indicated by the red line using the target sequence CAAGAAGACCGACATCCCCG. The gene-editing oligonucleotide GE-Oligo_02586 (green) with sequence CCTCACCCTCGACCTGGTCAAGAAGACCGAGCTGAGCCCATCTCCTGGATGACCTTTTCG was used for homology directed repair to create a deletion of 10 bp. Primers around the deletion (02586Fw: CTCACCCTCGACCTGGTCA and 02586Rv: CGAAAAGGTCATCCAGGAGA) were used for PCR verification. Expected band sizes are shown below each PCR product. GeneRuler 1 kb Plus DNA Ladder (Thermo Scientific) was run in lane L.

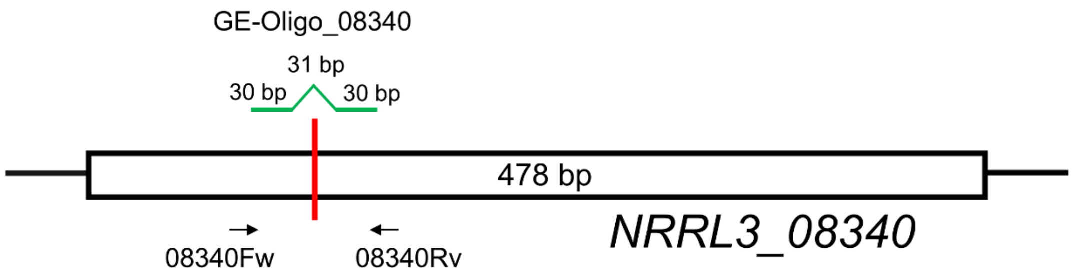

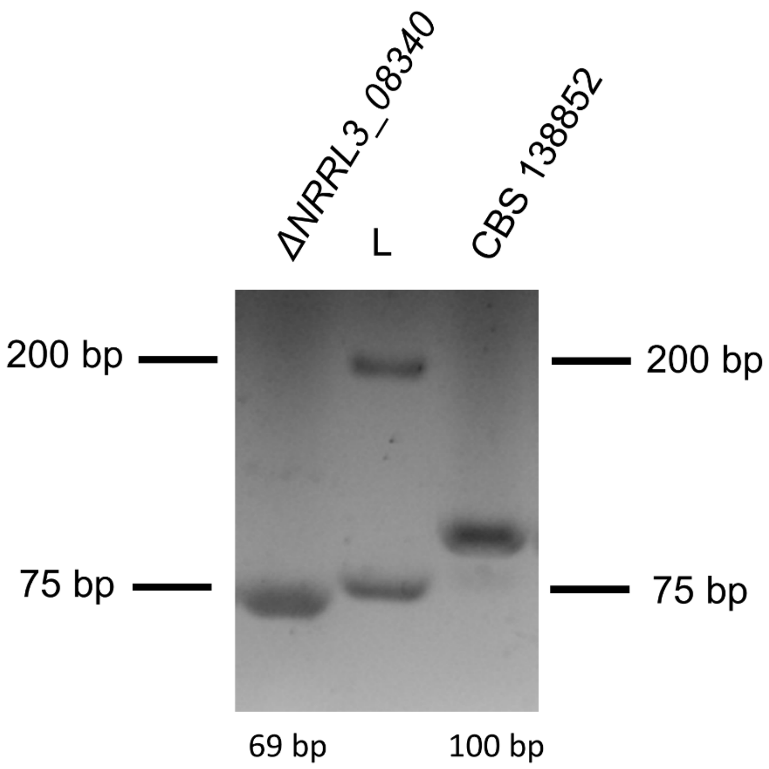

Figure S8. Diagram of mutant generation and verification method and gel used for verification of deletion in *NRRL3_08340*. CRISPR/Cas9 was used to create a double stranded break indicated by the red line using the target sequence AGGTATCGCCAACGACGGCG. The gene-editing oligonucleotide GE-Oligo_08340 (green) with sequence GCTCCACGAAACCCTCTTCGACGAGGGTATTACGTCGATAGAGCCTTGGCTCAAGGATCC was used for homology directed repair to create a deletion of 10 bp. Primers around the deletion (08340Fw: TTGACCAGCTCCACGAAA and 08340Rv: CTGGATCCTTGAGCCAAG) were used for PCR verification. Expected band sizes are shown below each PCR product. GeneRuler 1 kb Plus DNA Ladder (Thermo Scientific) was run in lane L.

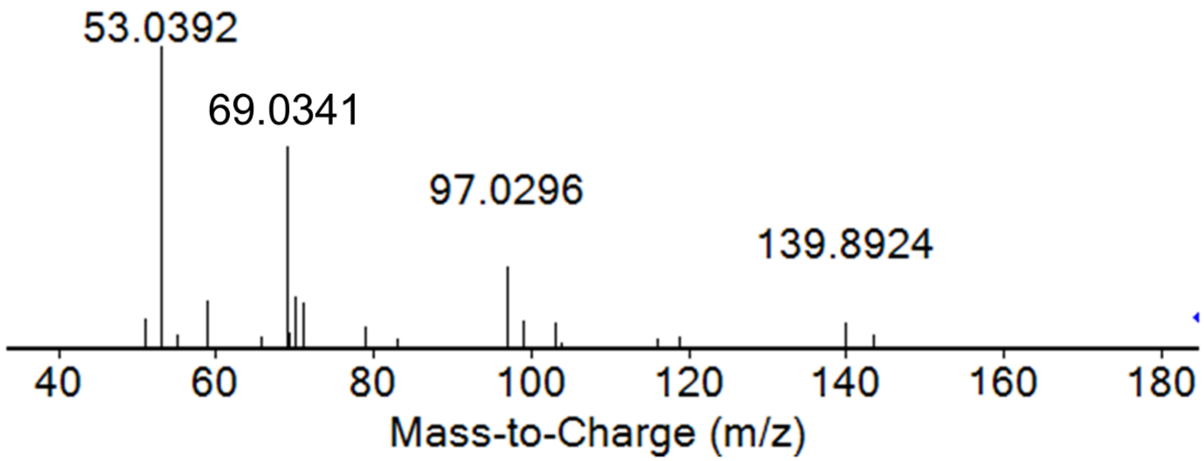

Figure S9. MS/MS fragment spectrum of the accumulated intermediate in the Δ*cmcA* mutant with mass 185.0102 in negative mode.

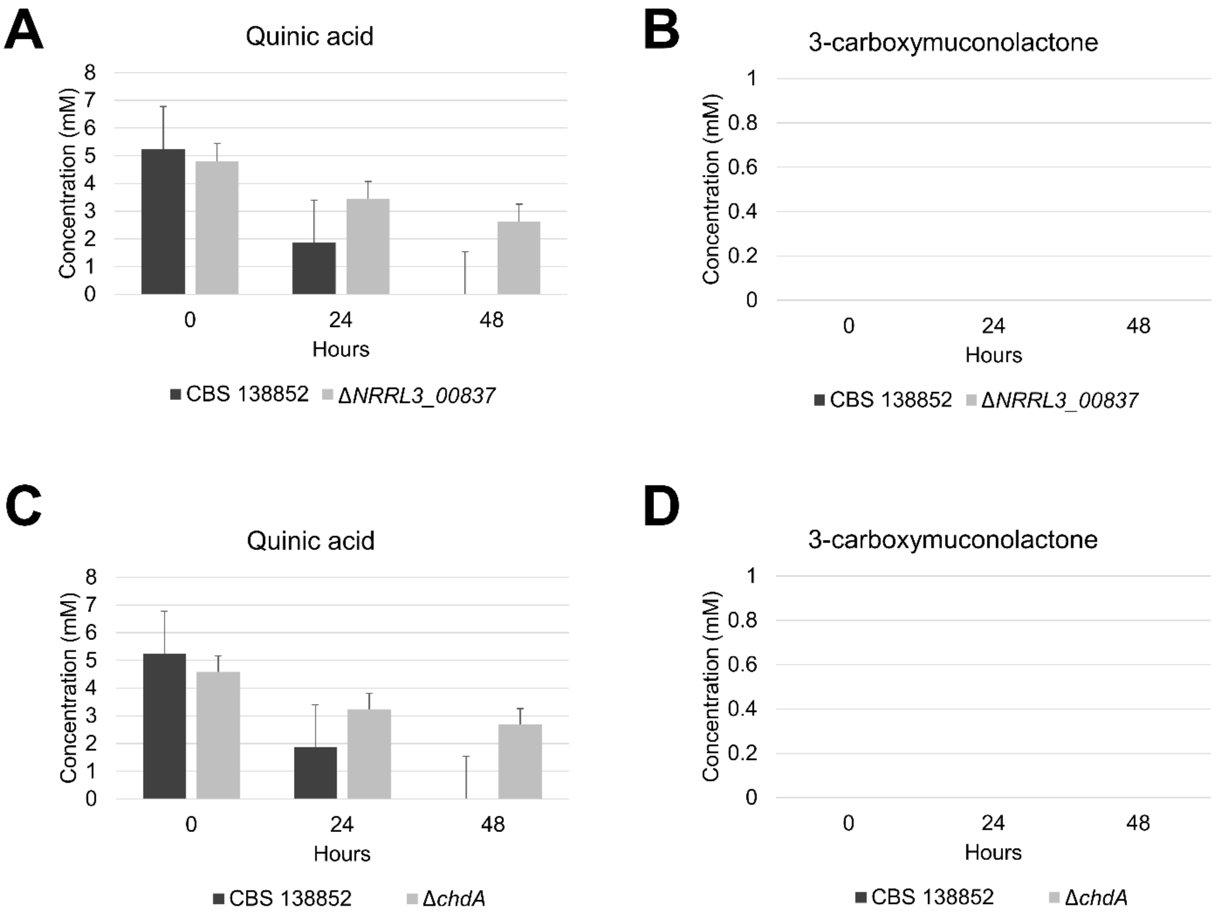

Figure S10. Accumulation of 3-carboxymuconolactone not detected in 3,4-DHB pathway mutants in minimal media with quinic acid as sole carbon source using LC-MS. (A) Consumption of quinic acid and (B) accumulation of 3-carboxymuconolactone in parent and Δ*NRRL3_00837* strains; (C) consumption of quinic acid and (D) accumulation of 3-carboxymuconolactone in parent and Δ*chdA* strains. Data from LC-MS in negative mode.

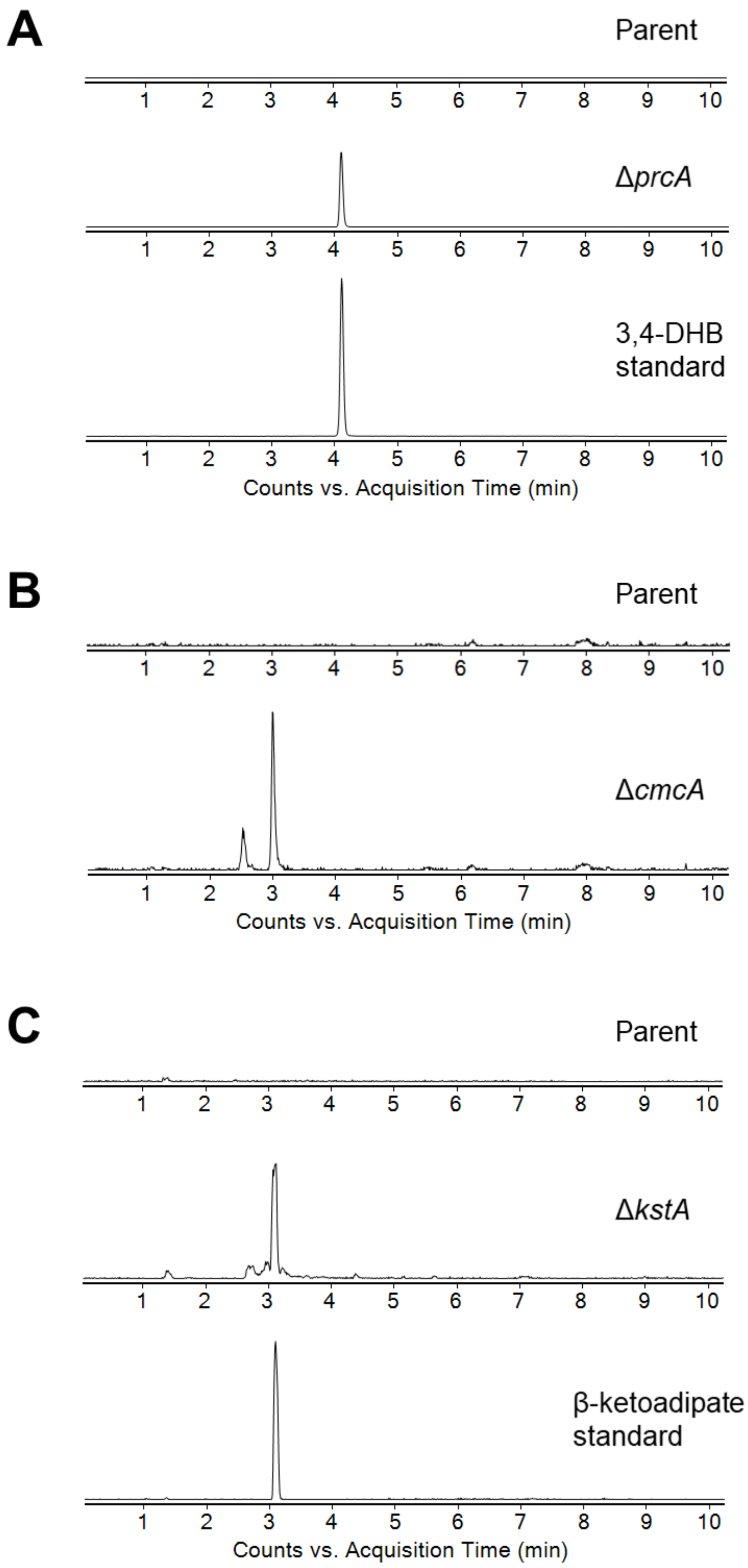
A
Parent
1 2 3 4 5 6 7 8 9 10
ΔprcA
1 2 3 4 5 6 7 8 9 10
3,4-DHB
standard
1 2 3 4 5 6 7 8 9 10
Counts vs. Acquisition Time (min)
B
Parent
1 2 3 4 5 6 7 8 9 10
ΔcmcA
1 2 3 4 5 6 7 8 9 10
Counts vs. Acquisition Time (min)
C
Parent
1 2 3 4 5 6 7 8 9 10
ΔkstA
1 2 3 4 5 6 7 8 9 10
β-ketoadipate
standard
1 2 3 4 5 6 7 8 9 10
Counts vs. Acquisition Time (min)

Figure S11. Extracted ion chromatograms of intermediates accumulated in the Δ*prcA*, Δ*cmcA*, and Δ*kstA* mutants. (A) Δ*prcA* mutant 48 hours after transfer to media with quinic acid as sole carbon source (top) and a standard of 3,4-dihydroxybenzoic acid (bottom). (B) the Δ*kstA* mutant 48 hours after transfer to media with quinic acid as sole carbon source (top) and a standard of β-ketoadipate (bottom). Samples were run in negative mode (A) and positive mode (B). Chromatograms are one of triplicate samples with nearly identical results.

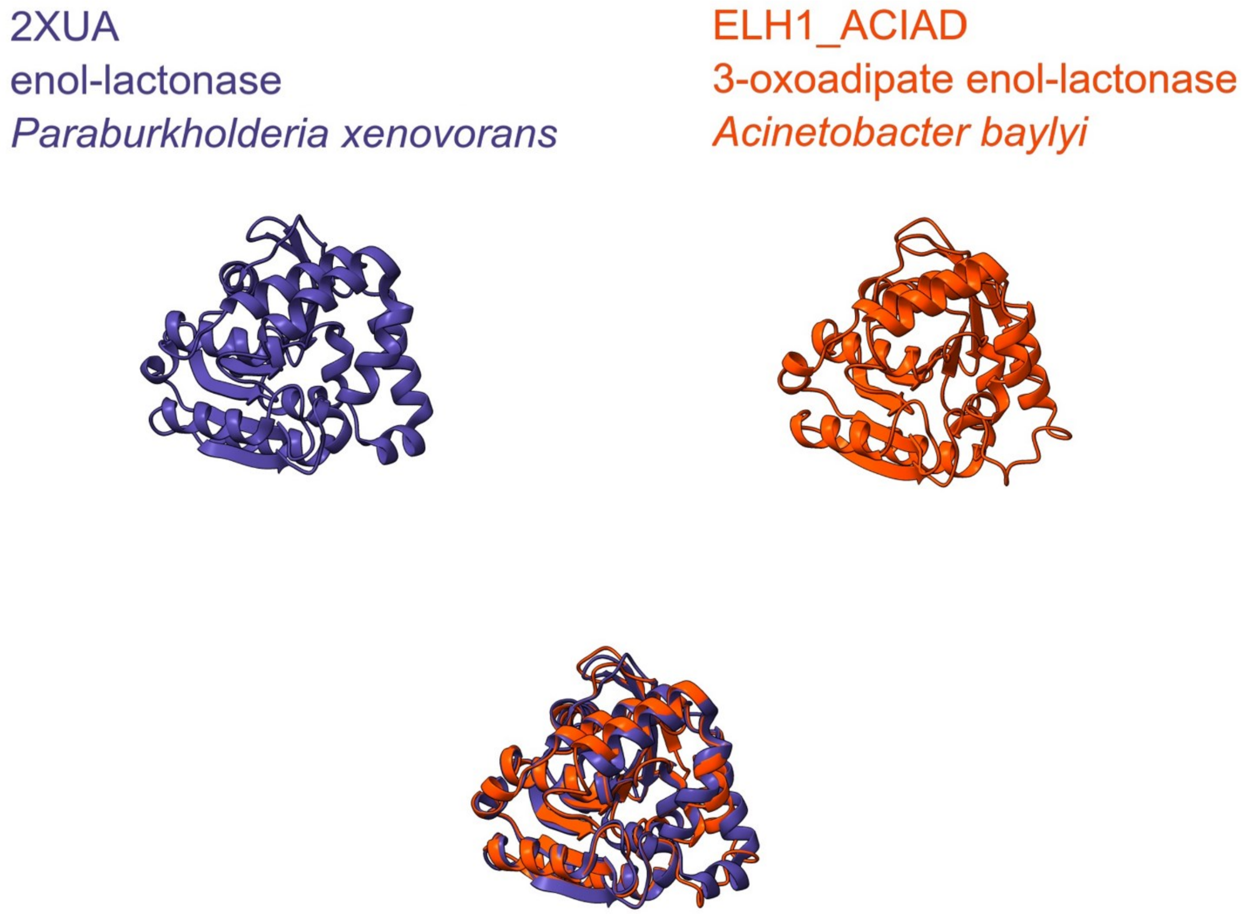

Figure S12. *Paraburkholderia xenovorans* enol-lactonase crystal structure (blue, Protein Data Bank ID 2XUA) compared to the *Acinetobacter baylyi* ELH1_ACIAD 3-oxoadipate enol-lactonase structure predicted by AlphaFold (red).

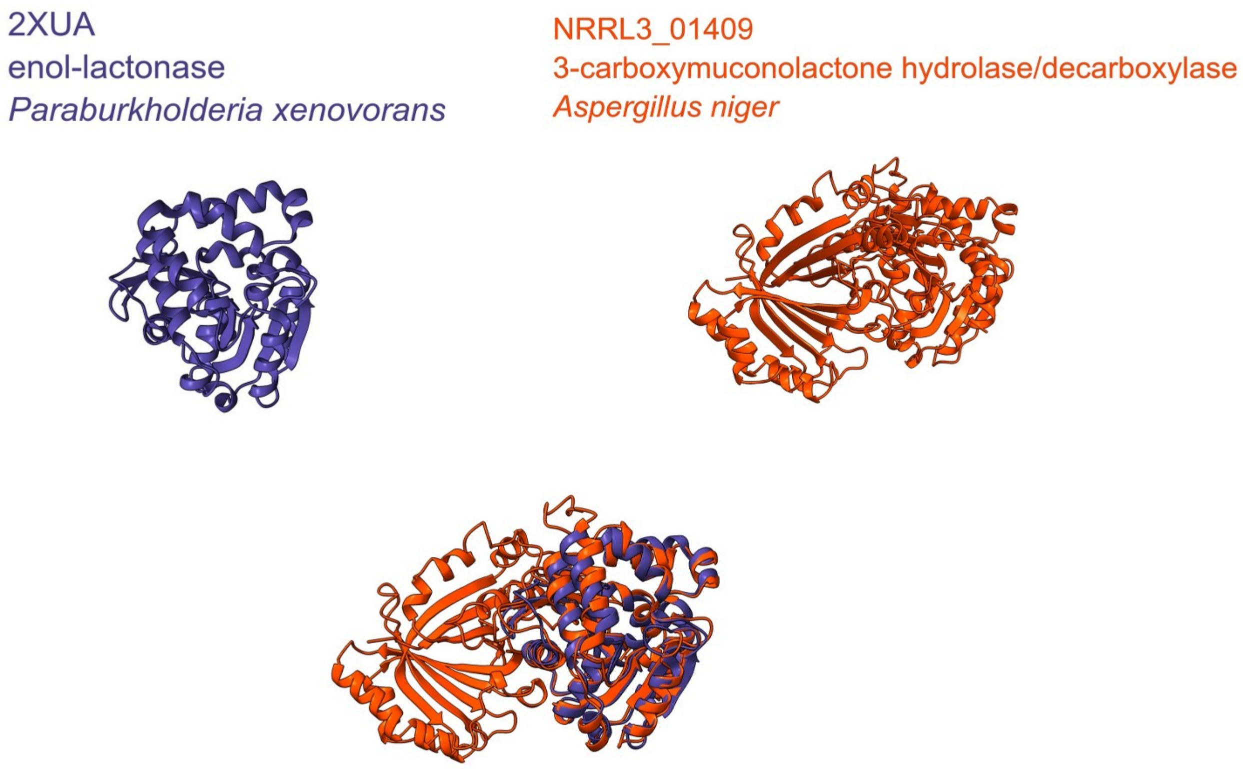

Figure S13. *Paraburkholderia xenovorans* enol-lactonase crystal structure (blue, Protein Data Bank ID 2XUA) compared to the *Aspergillus niger* NRRL3_01409 3-carboxymuconolactone hydrolase/decarboxylase structure predicted by AlphaFold (red).
